# LLM-Generated Lay-Language Protocols for Molecular Tumor Board Patients: Evaluation of Quality and Clinical Usability

**DOI:** 10.2196/99136

**Published:** 2026-07-23

**Authors:** Tabea Margareta Grace Pakull, Noëlle Bender, Sven Benson, Anke Fleischhauer, Mohammad Alsara, Tanja Gromke, Thomas Hilser, Katharina Kaminski, Michael Pogorzelski, Nicola Prasuhn, Vivian Rosery, Dirk Schadendorf, Martin Schuler, Marcel Wiesweg, Gregor Zaun, Peter Alexander Horn, Christoph Matthias Friedrich, Ina Pretzell

**Affiliations:** 1 Institute for Transfusion Medicine, Essen University Hospital, Hufelandstraße 55, Essen, North Rhine-Westphalia, 45147, Germany, 49 2017234201; 2 Department of Computer Science, Dortmund University of Applied Sciences and Arts, Dortmund, North Rhine-Westphalia, Germany; 3 Department of Human-Centered Computing and Cognitive Science, Social Psychology, University of Duisburg-Essen, Duisburg, North Rhine-Westphalia, Germany; 4 Centre for Translational Neuro- and Behavioral Sciences (C-TNBS), Institute for Medical Education, Essen University Hospital, Essen, North Rhine-Westphalia, Germany; 5 West German Cancer Center (WTZ), Essen University Hospital, Essen, North Rhine-Westphalia, Germany; 6 Department of Medical Oncology, West German Cancer Center (WTZ), Essen University Hospital, Essen, North Rhine-Westphalia, Germany; 7 Patient Partner, Patient Advisory Board, West German Cancer Center (WTZ), Essen University Hospital, Essen, North Rhine-Westphalia, Germany; 8 NCT West, National Center for Tumor Diseases (NCT), Essen, North Rhine-Westphalia, Germany; 9 Department of Dermatology, West German Cancer Center (WTZ), Essen University Hospital, Essen, North Rhine-Westphalia, Germany; 10 Institute for Medical Informatics, Biometry and Epidemiology (IMIBE), Essen University Hospital, Essen, North Rhine-Westphalia, Germany

**Keywords:** large language models, natural language processing, artificial intelligence, AI, precision medicine, lay language, Molecular Tumor Board, German, health literacy, evaluation, usability

## Abstract

**Background:**

Molecular Tumor Boards (MTBs) generate highly technical recommendations. The language used in their protocols is rarely accessible to patients. Lay-language patient protocols could support patient-clinician communication, yet manual production is difficult to sustain in high-volume oncology settings. Large language models (LLMs) may offer scalable drafting assistance, yet clinical usability remains largely uninvestigated under real-world deployment constraints. Existing evaluations rely predominantly on synthetic data or closed-source models that are incompatible with strict data protection requirements.

**Objective:**

This study evaluated whether open-weight LLMs can provide clinically usable drafting support for German MTB patient protocols under real-world deployment constraints and developed a transferable evaluation framework for patient-facing text generation.

**Methods:**

Eight open-weight LLMs were evaluated under zero-shot (A1) and one-shot (A2) prompting with constrained decoding, which ensures section-schema compliance. Automatic evaluation used ROUGE-1 (Recall-Oriented Understudy for Gisting Evaluation), BERTScore-F1 (Bidirectional Encoder Representations From Transformers Score), Wiener Sachtextformel version 4, and DistilBERT (Distilled Version of Bidirectional Encoder Representations From Transformers)–based complexity using a corpus of 316 MTB protocols and 47 expert-written patient protocols. For expert evaluation, 7 medical oncologists evaluated 50 protocols from the best-performing model across 3 International Organization for Standardization 9241‐11 usability dimensions using fine-grained error annotation, perceived postediting effort (PPEE), and net promoter score. Critical errors were defined as bearing the risk of patient harm.

**Results:**

Llama-3.3-70B-Instruct achieved the strongest automatic performance. Across models, A2 significantly improved most automatic metrics compared to A1. However, expert usability evaluation of Llama-3.3-70B-Instruct showed the opposite picture: the proportion of protocols containing at least 1 critical error doubled under A2 (10/25, 40% vs 5/25, 20%) compared with A1, and the dominant error type shifted from language (40/108, 37%) errors to factual errors (69/145, 48%). Overall, 16% (230/1420) of the annotated paragraphs contained errors. Median PPEE was 2 (IQR 2.0‐3.0; low), and median net promoter score was 7 (IQR 5.0-9.0). Detractors (46/100, 46%) outweighed promoters (29/100, 29%), which suggests hesitation toward routine adoption. These differences in expert evaluation between A2 and A1 were directionally consistent but did not reach individual statistical significance for the paired samples (n=25).

**Conclusions:**

Prompting strategies that improve automatic metrics can simultaneously increase the number of critical errors. Surface-level metric gains were, therefore, insufficient proxies for clinical safety. This was observed as a consistent directional pattern for a single model, but generalization to other models remains to be investigated. Nonetheless, the low paragraph-level error rate and favorable PPEE suggest that structured open-weight LLM generation may be a useful drafting support in a clinician-supervised setting. The proposed evaluation framework provides a text-quality-focused basis for future assessment of patient-facing LLM applications in real-world clinical settings.

## Introduction

Effective clinician-patient communication is fundamental to patient-centered care and shared decision-making [1], with demonstrated benefits for patient understanding, treatment adherence, and clinical outcomes [2]. This challenge is particularly acute in oncology. Molecular Tumor Boards (MTBs) [3] synthesize complex genomic and molecular findings to formulate individualized therapy recommendations for patients with advanced malignancies. Their recommendations are depicted in highly technical protocols that are largely inaccessible to lay readers. Written, lay-language summaries of MTB outcomes could support patient participation in treatment decisions and improve transparency in precision oncology care, yet their manual preparation is time-consuming and difficult to sustain in high-volume clinical environments. Automated drafting assistance for oncologists, as the intended users of such systems, is therefore a practically relevant prospect.

Large language models (LLMs) have attracted considerable interest for clinical communication tasks [4,5]. Their capacity for instruction-following and fluent text generation with minimal task-specific adaptation [6] positions them as candidate tools for lay-language protocol drafting. However, integration into clinical workflows remains constrained by well-documented concerns regarding factual accuracy, potential for harm, and output bias [5,7]. In oncology, clinician oversight of generated content is therefore structurally required rather than optional.

Two additional constraints narrow the practical design space. First, most published evaluations have focused on closed-source models [5] such as GPT-4 (OpenAI) [8]. These models are difficult to deploy for real patient data under current German and European data protection requirements. Therefore, open-weight alternatives that support on-premise deployment are the only viable option for most settings. Second, most existing studies were conducted on synthetic or deidentified data [4] and therefore offer limited relevance to operational clinical practice. Evaluation on real patient data under realistic hardware constraints remains an exception.

Another practical requirement is output conformity to institutional standards. Structured generation [9] addresses this by constraining fixed structural elements via predefined schemas and has been shown to improve format adherence and reduce hallucination rates in clinical note generation [10].

Our preliminary work [11] evaluated LLM-generated patient protocols from 4 MTB protocols using a single open-weight model with section-wise prompting. This approach led to narratively incoherent outputs across sections. Additionally, an LLM-as-a-judge approach proved insufficient to distinguish safe simplifications from factual errors, and a single-reviewer assessment without clinical expertise is methodologically inadequate for quality assurance in this setting. These findings identified 3 methodological requirements for a more rigorous follow-up study: structured generation for schema compliance, multimodel evaluation under realistic deployment constraints, and expert-led evaluation using a clinically grounded error taxonomy. The central challenge in this setting was not merely the generation of fluent lay-language communication, but to determine whether such outputs are clinically usable and safe as clinician-supervised drafting assistance.

**Figure 1.**
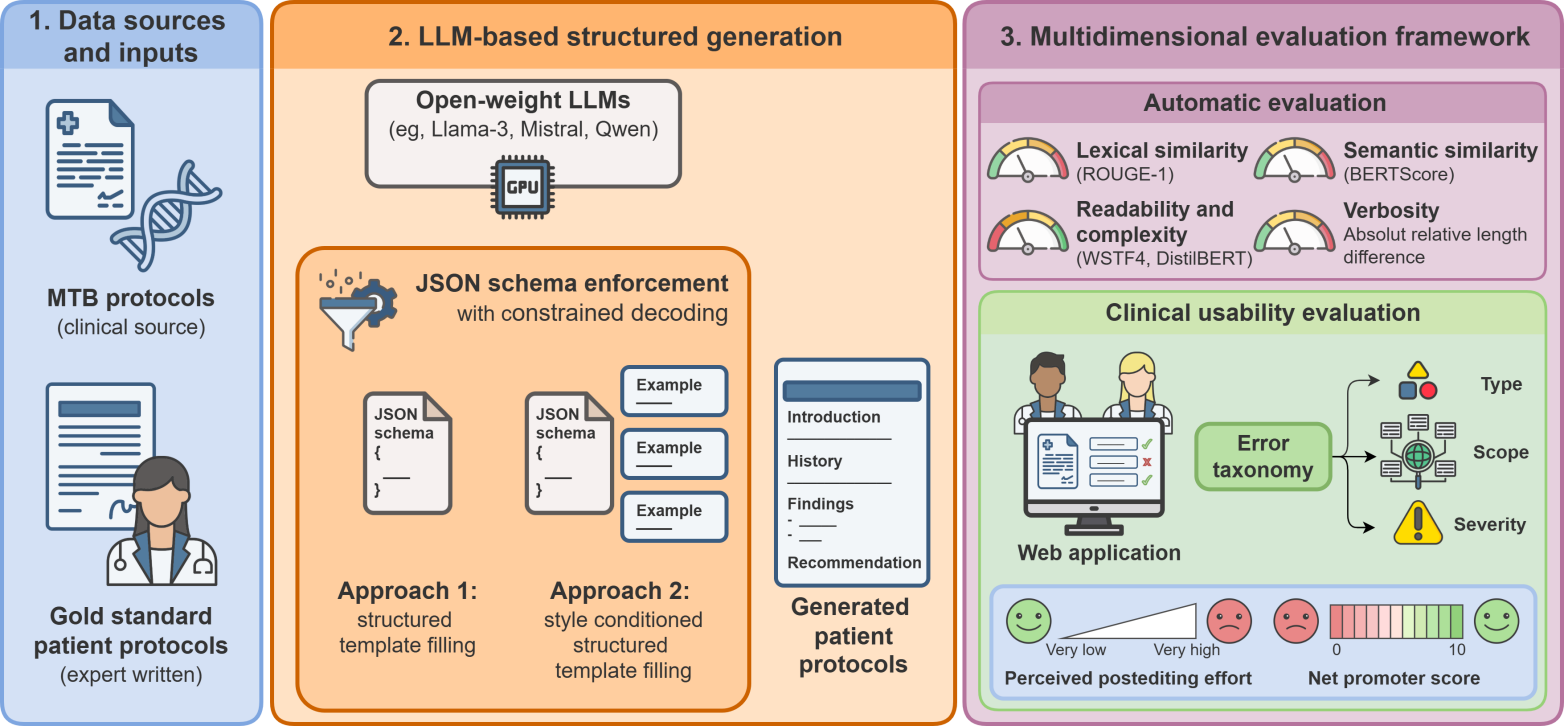
Study design overview showing data sources (MTB protocols and expert-written patient protocols), LLM-based structured generation under zero-shot (A1) and style-conditioned one-shot (A2) prompting, and a multidimensional evaluation framework assessing automatic metrics and clinical usability. BERTScore: Bidirectional Encoder Representations From Transformers Score; DistilBERT: Distilled Version of Bidirectional Encoder Representations From Transformers; LLM: large language model; MTB: Molecular Tumor Board; ROUGE: Recall-Oriented Understudy for Gisting Evaluation; WSTF4: Wiener Sachtextformel version 4.

This study addresses these requirements through 3 contributions. An overview of this study’s design is shown in Figure 1. First, it introduces a transferable multilevel evaluation framework operationalizing clinical usability across the 3 International Organization for Standardization (ISO) 9241‐11 [12] dimensions of effectiveness, efficiency, and satisfaction. Second, it evaluates a diverse panel of 8 open-weight LLMs under zero-shot and style-conditioned one-shot prompting. Third, it characterizes the relationship between prompting strategy, automatic metric performance, and expert-assessed clinical quality, and examines whether improvements in automatic metrics correspond to expert-assessed clinical quality. Taken together, these contributions serve 2 aims: introducing a clinical usability evaluation framework for lay language generation and determining the clinical usability of LLM-generated patient protocols under real-world deployment constraints.

## Methods

### Data

**Figure 2.**
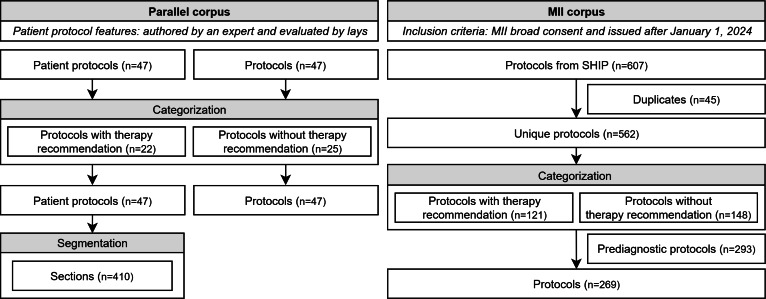
PRISMA flow diagram of the corpus construction process. MII: medical informatics initiative; PRISMA: Preferred Reporting Items for Systematic Reviews and Meta-Analyses; SHIP: smart hospital information platform.

Two German corpora of MTB and patient protocols were used in this study. Figure 2 shows the corpus construction process, and Table 1 summarizes its statistics.

A parallel corpus of 47 MTB protocols and expert-written patient protocols served as the gold standard for evaluation. The patient protocols were authored by an experienced oncologist specialized in precision oncology who leads an institutional MTB at the West German Cancer Center (WTZ) in Essen, Germany. The language and structure of the protocols align with a guideline developed by communication specialists, medical didacts, and the patient advisory board of the WTZ to ensure audience-appropriate language. A subset of the patient protocols (n=33) was evaluated in the MyCODE study [13], which supports their suitability as gold-standard references through positive assessments by lay readers and clinicians.

Protocols were categorized into protocols with therapy recommendation (n=22), where actionable molecular findings supported targeted therapy recommendations, and protocols without therapy recommendation (n=25), where no actionable molecular alterations were identified. These 2 case types follow distinct section schemas. Protocols with therapy recommendations were longer, as they contained more sections than protocols without therapy recommendations, primarily due to additional explanations of individual findings and supporting literature.

Patient protocols were manually segmented into 410 sections (227 with therapy recommendation and 183 without therapy recommendation) for reference-based evaluation.

For large-scale evaluation, 607 MTB protocols were exported via Fast Healthcare Interoperability Resources export from the smart hospital information platform, restricted to patients with Medical Informatics Initiative broad consent issued after January 1, 2024, and before September 25, 2025. After deduplication to remove overlaps with the parallel corpus, 562 unique MTB protocols were retained. A further 293 protocols were removed because they were not intended for patient communication. These documents only record an internal request for further diagnostic work and contain no findings or therapy recommendations to convey to patients. Finally, 269 protocols remained (with therapy recommendation, n=121 and without therapy recommendation, n=148).

**Table 1.** Descriptive corpus statistics for the parallel and MII^a^ corpora.

	Documents, n (%)	Sections, n (%)	Words per documents		
			Mean (SD)	Median (IQR)	Minimum-Maximum
Parallel					
Protocols	47 (100)	N/A^b^	881.8 (264.1)	849.0 (694.0-1097.0)	397‐1493
w/ TR^c^	22 (46.8)	N/A	1010.0 (270.4)	996.0 (809.3-1246.0)	582‐1493
w/o TR^d^	25 (53.2)	N/A	768.9 (203.3)	735.0 (642.0-916.0)	397‐1112
Patient protocols	47 (100)	410 (100)	558.0 (264.7)	464.0 (312.0-736.5)	229‐1221
w/ TR	22 (46.8)	227 (55.4)	780.9 (185.4)	736.5 (681.0-877.3)	464‐1221
w/o TR	25 (53.2)	183 (44.6)	361.9 (136.2)	319.0 (273.0-432.0)	229‐788
MII					
Protocols	269 (100)	N/A	1055.6 (340.4)	1021.0 (777.0-1277.0)	447‐2130
w/ TR	121 (45.0)	N/A	1077.5 (331.1)	1065.0 (831.0-1301.0)	468‐2113
w/o TR	148 (55.0)	N/A	1037.7 (347.9)	947.0 (773.3-1248.8)	447‐2130

a MII: medical informatics initiative.

b N/A: not applicable.

c w/ TR: protocols with therapy recommendation.

d w/o TR: protocols without therapy recommendation.

### Patient Protocol Generation

#### LLMs

As an initial step, multiple open-weight instruction-following LLMs were evaluated, served via vLLM [14] and queried using the OpenAI *Python* package [15], with inference parameters configured per official documentation (Multimedia Appendix 1). The models were selected based on 3 criteria: strong general-purpose generation capabilities, competitive multilingual coverage including German, and the feasibility of deployment on a single NVIDIA A100 (80 GB). Models exceeding 70B parameters were executed in 4-bit AWQ (activation-aware weight quantization) [16], except for the OpenAI models, which were natively released in MXFP4 (microscaling floating point 4) quantization [17]. The evaluated models deliberately span high-capacity state-of-the-art baselines and efficient small- to medium-sized models to quantify quality-efficiency trade-offs under realistic hardware constraints. These are summarized in Table 2.

This study was designed and reported in accordance with the TRIPOD-LLM (Transparent Reporting of a Multivariable Prediction Model for Individual Prognosis or Diagnosis) [18] guideline for studies evaluating LLMs in health care.

**Table 2.** Overview of the open-weight instruction-tuned LLMs^a^ used for evaluation.

Creator	Model	Short name	Parameter count	Reasoning
OpenAI	gpt-oss-120b [19]	G-120B	120B	Yes
OpenAI	gpt-oss-20b [19]	G-20B	20B	Yes
Meta	Llama-3.3-70B-Instruct [20]	L-70B	70B	No
Meta	Llama-3.1-8B-Instruct [20]	L-8B	8B	No
Mistral AI	Mistral-Large-Instruct-2411 [21]	M-123B	123B	No
Mistral AI	Magistral-Small-2509 [22]	M-24B	24B	Yes
Alibaba Cloud	Qwen3-Next-80B-A3B-Instruct [23]	Q-80B	80B	No
Alibaba Cloud	Qwen3-4B-Thinking-2507 [23]	Q-4B	4B	Yes

a LLM: large language model.

#### Structured Generation

MTB patient protocols follow defined section schemes, and structural malformation errors have been shown to have a significantly negative impact on clinical evaluation scores [24]. Two straightforward approaches for enforcing schema-compliant protocol generation were considered and rejected. Section-wise prompting, as used in our preliminary work, generates each section independently and cannot guarantee narrative coherence across the full protocol. Naive prompt-based template filling, where a protocol template is provided in the prompt and the model is instructed to complete it, proved brittle in pilot runs, as it produced altered headers, superfluous sections, and omitted mandatory fields.

Structured generation addresses both limitations by enforcing schema compliance at the token level through constrained decoding. Rather than instructing the model via natural language, constrained decoding, implemented through llguidance [25], mathematically prevents the model from generating any output that violates a predefined format. Separate JSON schemas were defined for protocols with and without therapy recommendation. As fixed structural elements are guaranteed by the schema, clinician review is limited to dynamic content sections only. This minimizes the oversight burden. The final protocols were rendered from the structured model output using a Jinja2 [26] template.

#### Experiments: Comparison of Zero-Shot and One-Shot Approach

Comparison of zero-shot and one-shot approaches was a key component of the analysis. As the available data precluded fine-tuning, both approaches used prompt-based control, which mirrors the constraints common in hospital settings. Complete prompt templates and schemas are provided in Multimedia Appendix 2.

Regarding approach 1, structured template filling (A1) used zero-shot, schema-driven generation without examples. A system prompt specified persona, rules, and style constraints. The user prompt contained the generation instruction, short field-level instructions injected via the JSON schema, and the MTB protocol as source content.

Regarding approach 2, style-conditioned structured template filling (A2) extended A1 with one-shot, style-conditioned generation, motivated by in-context learning [6]. The same schema and structured decoding were used, with one expert-written example injected per dynamic field, following the field instructions to anchor tone, lexical choices, and level of detail to institutional practices.

### Evaluation

#### Automatic Evaluation

Given the limited gold standards and the requirement for German-suitable metrics, automatic evaluation combined reference-based fidelity metrics with reference-free readability and complexity measures. Reference-based metrics were computed for the 410 protocol sections by aligning the generated JSON fields with the corresponding sections in the expert-written protocols. These reference protocols were written using a structured framework grounded in didactic and psychological principles, codeveloped by MTB physicians, psychologists, and the WTZ patient advisory board [13]. Reference-based metrics should therefore be interpreted as measures of stylistic and structural proximity to this reference, not as absolute indicators of lay-language quality.

Lexical fidelity was assessed using ROUGE-1 (Recall-Oriented Understudy for Gisting Evaluation) [27], which measures the word-level overlap between generated and reference texts (0‐1, higher is better).

Semantic fidelity was assessed using BERTScore-F1 (Bidirectional Encoder Representations From Transformers Score) [28], which computes embedding-level similarity to capture paraphrases and conceptual overlap (0‐1, higher is better).

Readability metrics can be applied without gold standards because they rely solely on text features, such as sentence and word length. Readability was assessed using Wiener Sachtextformel version 4 (WSTF4) [29], a formula optimized for German, computed from the MS (percentage of words with 3 or more syllables) and the SL (average sentence length in words):


$$WSTF_{4}=0.2744\times MS+0.2656\times SL-1.1693$$

Scores of 4‐6 indicate very easy texts, 7‐10 well-understood, 10‐12 medium difficulty, and above 12 specialist-level; scores can additionally be interpreted as the approximate school grade level required for comprehension.

Text complexity was assessed using a DistilBERT (Distilled Version of Bidirectional Encoder Representations From Transformers) model fine-tuned on German texts [30]. The model yields scores on a 1‐7 scale, with lower values indicating simpler texts.

The differences in verbosity were quantified as the relative length differences between the generated and gold standard sections in words. A verbosity score of 1−|Δlength| was used, where higher values indicate lower deviation. This score is a proximity measure with a range of (−∞, 1], and no clipping or normalization was applied. All verbosity results are reported as medians, which remain robust against the extreme values caused by overgeneration.

#### Error Taxonomy

Automated metrics provide a scalable proxy for linguistic fluency but are notoriously brittle in clinical contexts due to their inability to capture medical safety implications or the specific error types inherent in LLM-generated outputs.

Rigorous evaluation of patient protocols requires a taxonomy that covers factual correctness, communicative adequacy, and patient safety. Established frameworks such as the Physician Documentation Quality Instrument-9 [31] or SummEval [32] have been proven useful, yet both assign scores to quality criteria rather than pinpointing specific errors, a level of detail that more closely reflects the correction work clinicians actually perform. Error annotation frameworks proposed for summarization [33], translation [34], and clinical documentation [10] offer finer-grained analysis but treat any addition beyond the source document as an error or hallucination. This assumption represents a fundamental mismatch for patient protocol generation, where explanatory additions are necessary to achieve health literacy alignment. Penalizing such elaborations would systematically underestimate quality and mischaracterize safe model behavior.

Therefore, a specific taxonomy was developed. The taxonomy proposed here classifies each identified issue along 3 dimensions: 4 error types (factual, omission, noise, and language), 2 contextual scopes (internal and external), and 3 severity levels (minor, major, and critical). Reports that contain at least one critical error are rated as invalid and not acceptable for clinical use.

The scope dimension reflects the dual information sources of patient protocols: the factual content of the MTB protocol (internal) and the general medical background knowledge required to make the content comprehensible to lay readers (external). This distinction prevents the misclassification of clinically necessary explanatory additions as hallucinations or factual errors.

The four error types are defined as follows: (1) factual errors occur when the text contradicts either the source document (internal), such as incorrect diagnoses or therapy recommendations, or established medical knowledge (external), such as a misdefined biomarker; (2) omission errors occur when relevant information is absent. Internal omissions cover patient-specific details from the source document, and external omissions cover missing background explanations necessary for lay understanding; (3) noise errors occur when a text contains unnecessary or misleading content. Internal noise comprises redundant details from the source document, and external noise refers to unrelated medical information that dilutes key messages; and (4) language errors are linguistic or stylistic issues (eg, grammar, syntax, or phrasing) that do not affect factual content but may reduce readability, professionalism, or patient trust.

Severity reflects the potential impact on patient understanding and safety. Minor errors involve terminological or stylistic inaccuracies with no safety implications. Major errors substantially impair lay comprehension or distort key medical facts. The patient may misunderstand content, yet no direct harm or dangerous consequence is likely to result. Critical errors carry direct patient safety implications and risk harm through misinformation, such as incorrect therapy instructions or the omission of essential safety warnings. Annotators must assign the highest applicable severity level.

#### Expert Evaluation

Following best practices from machine translation human evaluation research, only domain experts participated in the manual evaluation, given the interpretive complexity of clinical content. Seven board-certified medical oncologists or hematologists from the WTZ served as annotators, with direct experience in complex MTB cases constituting relevant domain expertise.

A stratified random sample of 25 MTB cases was drawn from the parallel corpus (20 with therapy recommendations and 5 without therapy recommendations). Priority was given to cases with therapy recommendations to ensure a focus on high-complexity reports with more dynamic fields and greater error risk. Consequently, expert-level characterization of protocols without therapy recommendations is more limited. To avoid distortion, there was no “human-in-the-loop” before the expert evaluation. Using the top-performing model from the automatic evaluation, paired A1 and A2 outputs yielded 50 unique protocols. Restricting expert evaluation to a single model was a deliberate choice, as including multiple models was not feasible given the annotation workload, and the primary goal was to compare generation approaches rather than models. Each protocol was independently rated by 2 clinicians, for a total of 100 ratings. As no consensus discussion or Delphi reconciliation was performed, interannotator agreement was quantified post hoc using Gwet agreement coefficient 1 (AC1), as described in the Statistical Analysis section.

A between-subject blinded design preserved ecological validity: each annotator saw each MTB protocol only once, and the generation conditions (A1 vs A2) were undisclosed. This mirrors anticipated routine deployment conditions, where each clinician reviews a given case only once and thereby avoids potential fatigue effects.

Clinical usability was assessed along the 3 ISO 9241‐11 usability dimensions of effectiveness, efficiency, and satisfaction using 3 complementary instruments.

1. Paragraph-level error annotation (effectiveness): annotators applied the error taxonomy described above at the paragraph level. They annotated error type, scope, and severity. This granular approach captures both specific error profiles and their potential clinical impact.
2. Perceived postediting effort (PPEE and efficiency): drawing on effort expectancy theory [35] and machine translation postediting research [36,37], clinicians rated the anticipated workload to bring each protocol to patient-ready quality on a 5-point Likert scale (1=very low, 5=very high): “How much effort would be required to edit this protocol to make it suitable as a patient protocol?”
3. Net promoter score [38] (NPS and satisfaction): Clinicians rated their overall satisfaction using the NPS on a 0‐10 scale; they answered a single item: “How likely are you to recommend the AI-generated draft shown above to a colleague for creating a patient protocol?”

Together, these 3 instruments capture distinct and decision-relevant aspects of usability: error annotation reflects objective output quality, PPEE proxies operational integration burden, and NPS captures broader clinical acceptability.

A dedicated web interface built using Streamlit [39] supported expert evaluation. Hosted within the hospital network and accessible via institutional credentials, it comprised 3 components: a project overview describing this study and error taxonomy, the core evaluation interface, and a visual decision tree guiding annotators through taxonomy category assignment. The evaluation interface used a dual-pane layout. The left panel displayed the original MTB protocol; the right panel presented the generated patient protocol divided into dynamic sections. Annotators first read the full protocol and then assessed each dynamic section for errors. Multiple errors could be recorded per section with optional free-text comments. After completing section-level annotation, annotators rated the PPEE and NPS for the full document. All annotations were saved as JSON files for subsequent analysis.

Annotators took part in a training procedure that included an online session, 2 written reference documents, and 5 practice protocols not part of the final evaluation set. During the online session, this study’s team walked through the taxonomy and the interface and answered annotators’ questions directly. One reference document explained the error taxonomy with definitions and worked examples. The other described the evaluation interface. A decision tree for step-by-step category assignment was embedded in both the interface and the taxonomy document and was available throughout annotation.

### Statistical Analysis

#### Automatic Evaluation

For each system (defined as LLM×prompting approach) and each metric, the results are summarized as the median with a 95% percentile bootstrap CI (5000 resamples, stratified by document).

System-level comparisons used pairwise tournament analysis to evaluate all system combinations head-to-head across metrics. For each pair, the proportion of instances in which system i outperformed system j was computed. The net win percentages (wins minus losses) yield an interpretable scale-independent performance ranking.

The primary inferential analysis compared the 2 prompting approaches (A2 vs A1) within each LLM using the Wilcoxon signed-rank test [40] on document-level paired differences (A2−A1). The effect size was quantified using the matched-pairs rank-biserial correlation r, where *r*=1 indicates perfect superiority of A2 and r=−1 indicates perfect inferiority. Benjamini-Hochberg correction [41] controlled the false discovery rate. All raw *P* values (8 LLMs×5 metrics) were pooled into one correction family per analysis domain. Adjusted *P* values below .05 are considered statistically significant.

All analyses were conducted across 3 prespecified subsets (overall, with therapy recommendation, and without therapy recommendation) to account for potential heterogeneity in protocol structure and vocabulary between document types.

Analyses were implemented in Python (version 3.12.11), using NumPy, pandas, SciPy, and statsmodels.

#### Expert Evaluation

Five effectiveness outcomes were captured at the document level: error count, severity-weighted error score (minor=1, major=2, and critical=3), and errors stratified by type, scope, and severity. Spearman rank-order correlation (ρ) quantified the association between the severity-weighted error score and expert ratings (NPS and PPEE). NPS was additionally analyzed using the standard 3-category classification (detractors: 0‐6, passives: 7‐8, and promoters: 9‐10).

For each approach and outcome, the results are reported as the median with a 95% percentile bootstrap CI (5000 resamples). Before the A1 vs A2 comparisons, error counts were averaged across the 2 raters to obtain a single case-level estimate.

The highly zero-inflated distribution of paired differences (eg, 17/25, 70%, identical outputs in the noise category) made the Wilcoxon signed-rank test inappropriate. Instead, an exact binomial sign test [42] on nontied pairs assessed pairwise approach preference, with Cohen g reported as the effect size measure. Benjamini-Hochberg correction controlled the false discovery rate across all outcomes within each analysis domain. Adjusted *P* values below .05 are considered statistically significant.

Interannotator agreement on error presence was evaluated using Gwet AC1 [43]. This metric was deliberately chosen to counter the highly imbalanced nature of the evaluation data, where the absence of errors constituted the overwhelming majority of the observations. In such skewed distributions, metrics such as Krippendorff α suffer from the prevalence paradox, severely underestimating reliability. All coefficients included bootstrapped 95% CIs (5000 resamples).

Analyses were implemented in Python (version 3.12.11), using NumPy, SciPy, pandas, and the irrCAC library.

### Ethical Considerations

The institutional ethics committee of the Medical Faculty, University of Duisburg-Essen, approved this study, with no ethical or legal concerns raised (reference: 24‐12093-BO; June 16, 2025). Data use was restricted to MTB protocols from patients who consented within the Medical Informatics Initiative broad consent; no patient recontact or intervention occurred. All data processing, model inference, and evaluation tools were hosted on secure institutional servers within the University Hospital network to ensure regulatory compliance.

## Results

### Automatic Evaluation

**Figure 3.**
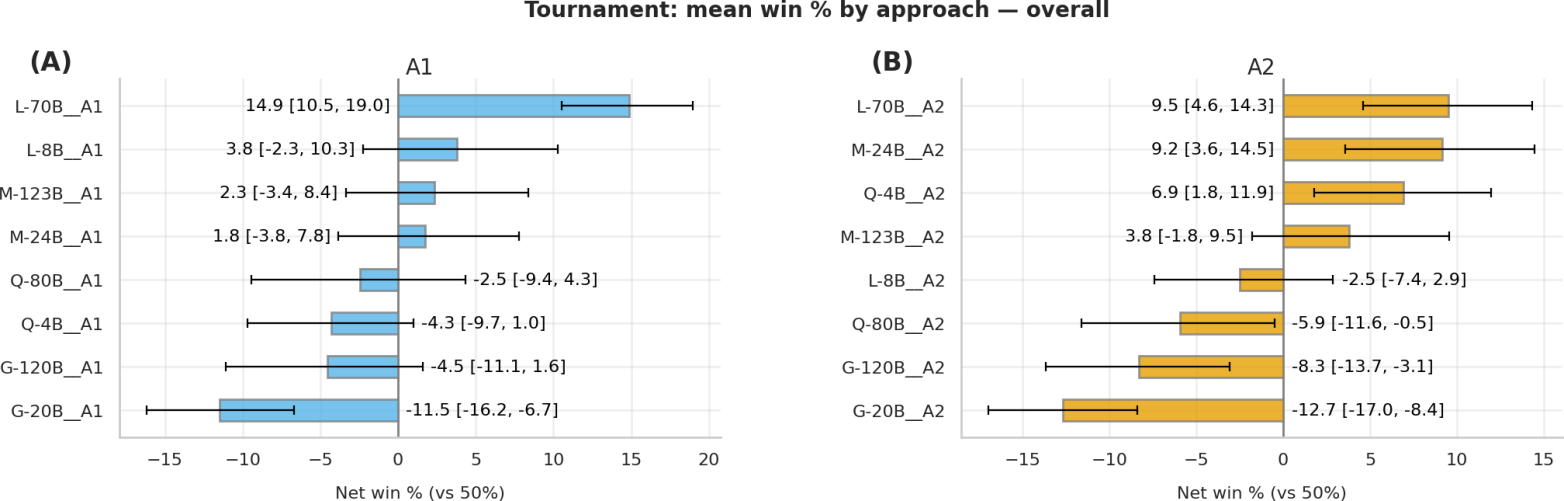
Tournament ranking across datasets for A1 (A, left) and A2 (B, right). Each bar represents the mean net win percentage of a given LLM against all other LLMs, computed relative to an overall tie baseline of 50% in a pairwise metric comparison. Error bars indicate 95% bootstrap CIs. LLM: large language model.

In the pairwise tournament (Figure 3), Llama-3.3-70B-Instruct (L-70B) achieved the highest net win percentage across all metrics in both approaches (+14.9 pp under A1 and +9.5 pp under A2), while gpt-oss-20b (G-20B) ranked the lowest (−11.5 pp under A1 and −12.7 pp under A2). In both with- and without-therapy-recommendation subsets, the pairwise tournament rankings were broadly consistent; however, in cases without therapy recommendation, Mistral-Large-Instruct-2411 (M-123B) marginally outranked L-70B in the combined (A1+A2) tournament, whereas L-70B ranked first in cases with therapy recommendation.

The median metric values (Table 3) corroborated these trends. Under A1, the highest-scoring system was L-70B, and the lowest-scoring was G-20B. Under A2, L-70B again achieved the highest fidelity scores (ROUGE-1: 0.67 and BERTScore-F1: 0.63). A2 yielded significantly higher ROUGE-1 and BERTScore-F1 scores than A1 across all 8 models (Table 4). Verbosity scores show that generated protocols deviated 21%‐33% from the reference length in A1; A2 reduced this to 18%‐28%. Verbosity improvements from A1 to A2 were statistically significant for 5 of the 8 models, but not for L-70B, Llama-3.1-8B-Instruct (L-8B), or gpt-oss-120b (G-120B). Across all sections, models, and approaches, the verbosity score had a median of 0.8 (IQR 0.4‐1.0). The minimum was −667, caused by a small number of pathologically long generations. As verbosity is reported as a median, these tail values did not affect the per-system scores.

The WSTF4 scores significantly improved from A1 to A2 for all models. The largest effects were observed for the models with the worst A1 scores: L-8B (*r*=0.53), G-120B (*r*=0.43), and G-20B (*r*=0.35). Under A2, L-70B, M-123B, and Magistral-Small-2509 (M-24B) fell marginally below the gold standard (10.73 and 10.72 vs 10.89). DistilBERT-based complexity assessments showed that all generated texts remained far from the gold standard (2.79) within a similar complexity range (4.10‐4.78), with no systematic differences between the approaches.

**Table 3.** Median automatic evaluation metrics across datasets for approaches 1 and 2. The gold standard row reports the reference free metrics for expert-written patient protocols. Medians were computed for dynamic sections only.

	ROUGE-1 ↑	BERTS-F1^a^ ↑	WSTF4^b^ ↓	Complexity^c^ ↓	Verbosity ↑
Gold standard	N/A^d^	N/A	10.89	2.79	N/A
Approach 1, median (IQR)					
G-120B	0.50 (0.26-1.00)	0.45 (0.20-1.00)	11.97 (8.25-13.90)	4.72 (1.93-5.21)	0.79 (0.38-1.00)
G-20B	0.40 (0.24-1.00)	0.39 (0.20-1.00)	11.93 (8.25-13.89)	4.36 (1.93-5.17)	0.70 (0.32-1.00)
L-70B	0.53 (0.25-1.00)	0.53 (0.22-1.00)	10.88 (8.25-13.12)	4.25 (1.93-4.98)	0.79 (0.36-1.00)
L-8B	0.40 (0.23-1.00)	0.48 (0.20-1.00)	11.67 (8.25-13.50)	4.10 (1.93-4.88)	0.67 (0.36-1.00)
M-123B	0.40 (0.25-1.00)	0.44 (0.21-1.00)	10.94 (8.25-13.04)	4.18 (1.87-5.15)	0.67 (0.37-1.00)
M-24B	0.42 (0.27-1.00)	0.40 (0.25-1.00)	10.92 (8.25-13.00)	4.34 (1.93-4.98)	0.68 (0.32-1.00)
Q-80B	0.47 (0.29-1.00)	0.43 (0.23-1.00)	11.31 (8.25-13.09)	4.78 (1.93-5.24)	0.76 (0.21-1.00)
Q-4B	0.40 (0.27-1.00)	0.43 (0.22-1.00)	11.68 (8.25-13.37)	4.39 (1.93-5.08)	0.78 (0.25-1.00)
Approach 2, median (IQR)					
G-120B	0.56 (0.32-1.00)	0.57 (0.27-1.00)	11.40 (8.25-13.15)	4.76 (1.91-5.19)	0.81 (0.42-1.00)
G-20B	0.55 (0.29-1.00)	0.53 (0.25-1.00)	11.41 (8.25-13.22)	4.64 (1.91-5.19)	0.81 (0.39-1.00)
L-70B	0.67 (0.37-1.00)	0.63 (0.36-1.00)	10.73 (8.25-12.52)	4.46 (1.92-5.09)	0.80 (0.44-1.00)
L-8B	0.56 (0.34-1.00)	0.56 (0.32-1.00)	11.00 (8.25-12.77)	4.34 (1.93-5.10)	0.75 (0.42-1.00)
M-123B	0.56 (0.33-1.00)	0.56 (0.33-1.00)	10.72 (8.25-12.82)	4.19 (1.89-5.13)	0.72 (0.38-1.00)
M-24B	0.63 (0.39-1.00)	0.59 (0.36-1.00)	10.72 (8.25-12.50)	4.34 (1.93-5.16)	0.76 (0.43-1.00)
Q-80B	0.57 (0.34-1.00)	0.56 (0.29-1.00)	10.92 (8.25-12.88)	4.71 (1.93-5.25)	0.82 (0.38-1.00)
Q-4B	0.61 (0.37-1.00)	0.59 (0.32-1.00)	11.14 (8.25-13.04)	4.34 (1.93-5.11)	0.82 (0.49-1.00)

a BERTS-F1: Bidirectional Encoder Representations From Transformers Score.

b WSTF4: Wiener Sachtextformel version 4.

c Complexity: DistilBERT (Distilled Version of Bidirectional Encoder Representations From Transformers)-based complexity.

d N/A: not applicable.

**Table 4.** Median paired differences in evaluation metrics between approaches (approach 2−approach 1) across datasets with effect sizes and *P* values.

LLM^a^	ROUGE-1			BERTS-F1^b^			WSTF4^c^			Complexity^d^			Verbosity		
	Δ	r	*P*	Δ	r	*P*	Δ	r	*P*	Δ	r	*P*	Δ	r	*P*
G-120B	0.06	0.48	.02	0.07	0.66	.001	−0.32	0.43	<.001	0.00	0.02	.73	0.04	0.31	.07
G-20B	0.03	0.63	.002	0.03	0.59	.003	−0.25	0.35	<.001	0.10	−0.34	<.001	0.10	0.57	.002
L-70B	0.03	0.77	<.001	0.02	0.72	.001	−0.02	0.15	.03	0.19	−0.53	<.001	0.00	−0.09	.66
L-8B	0.06	0.59	.002	0.05	0.54	.005	−0.50	0.53	<.001	0.21	−0.54	<.001	0.05	0.32	.07
M-123B	0.09	0.70	.001	0.11	0.76	<.001	−0.18	0.21	.002	−0.01	−0.01	.90	0.04	0.50	.006
M-24B	0.05	0.84	<.001	0.07	0.84	<.001	−0.28	0.41	<.001	0.08	−0.20	.003	0.09	0.63	<.001
Q-80B	0.00	0.80	<.001	0.00	0.82	<.001	−0.22	0.33	<.001	−0.02	0.28	<.001	0.06	0.62	.002
Q-4B	0.13	0.88	<.001	0.12	0.86	<.001	−0.33	0.41	<.001	−0.10	0.20	.002	0.10	0.63	<.001

a LLM: large language model.

b BERTS-F1: Bidirectional Encoder Representations From Transformers Score.

c WSTF4: Wiener Sachtextformel version 4.

d Complexity: DistilBERT (Distilled Version of Bidirectional Encoder Representations From Transformers)-based complexity.

**Figure 4.**
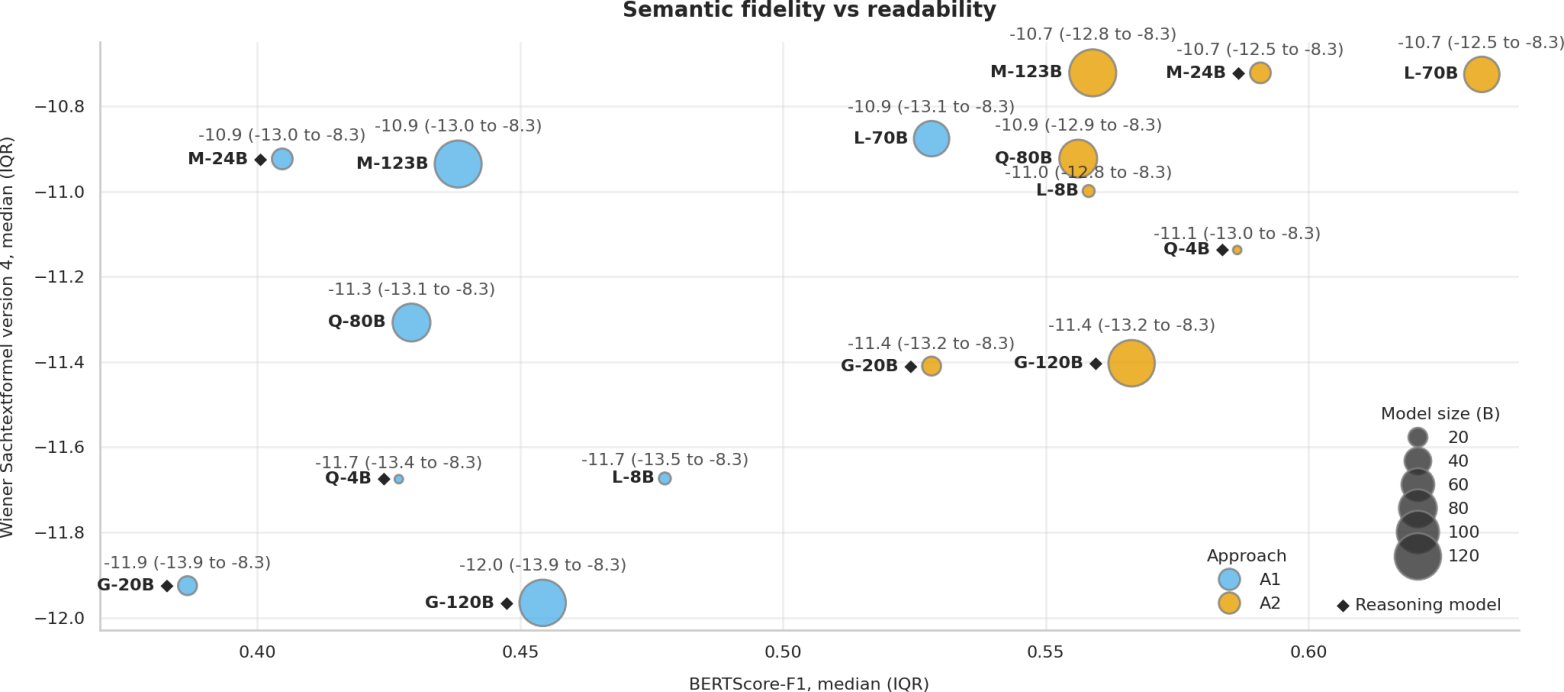
Comparison of semantic similarity (BERTScore) and readability (WSTF4) across prompting approaches (A1: blue and A2: orange). WSTF4 is inverted, such that higher values indicate higher readability. Marker size indicates model size, and reasoning capabilities are marked with a diamond. BERTScore: Bidirectional Encoder Representations From Transformers Score; WSTF4: Wiener Sachtextformel version 4.

In the fidelity-readability scatter plot (Figure 4), the A2 systems clustered toward higher BERTScore-F1 and lower WSTF4 values relative to their A1 counterparts. L-70B_A2 and M-123B_A2 showed the most favorable profiles, as they achieved a BERTScore-F1 ≥.59 and a WSTF4 ≥−10.73.

A1-to-A2 improvements in lexical and semantic fidelity were more pronounced in cases with therapy recommendation. However, higher fidelity does not necessarily mean improved quality. It only reflects a closer alignment with the reference. Median paired differences in ROUGE-1 and BERTScore were significant across models in cases with therapy recommendation, whereas median differences were near zero and nonsignificant for all 8 models in cases without therapy recommendation. WSTF4 improvements were significant in both case types for all models, except L-70B for cases without therapy recommendation. Detailed results per case type can be found in Multimedia Appendix 3.

Notably, neither the model parameter count nor the use of reasoning capabilities was associated with a consistent performance advantage across metrics.

### Clinical Usability

#### Effectiveness: Error Annotation

In 16% (230/1420) of annotated paragraphs, experts identified a total of 253 errors, which resulted in a median of 2.0 (IQR 1.5‐3.5) errors per protocol. By approach, A1 yielded a median of 2.0 (IQR 1.5‐2.5) errors per protocol and A2 a median of 2.5 (IQR 1.5‐3.5) errors per protocol. Median paired differences were *Δ*=0.0 (95% CI −0.5 to 2.0), with no significant differences observed in error counts between A1 and A2.

The proportion of protocols with ≥1 critical error was 20% (n=5) for A1 and 40% (n=10) for A2. The median of critical errors per protocol was 0.0 (IQR 0.0‐0.5) for A1 and 0.5 (IQR 0.0‐1.5) for A2. Major errors occurred at a median of 0.5 (IQR 0.0‐0.5) per protocol for A1 vs 1.0 (IQR 0.0‐1.5) for A2, while minor errors occurred at 1.0 (IQR 0.5‐1.5) for both A1 and A2. Median paired differences in error severity between approaches were *Δ*=0.0 (95% CI 0.0 to 1.0) for critical errors, *Δ*=0.5 (95% CI 0.0 to 1.0) for major errors, and Δ=−0.5 (95% CI −1.0 to 0.5) for minor errors.

Critical errors fell into 2 main categories. The largest group, with 24 annotations across 12 protocols, involved misjudgment of clinical relevance. This included providing explanations for therapeutically irrelevant alterations, omitting secondary options such as genetic counseling or trial referrals, and incorrectly characterizing the evidence base. These errors occurred under both prompting conditions, but they were more frequent under condition A2. The increase in critical errors under A2 was driven by exemplar contamination (18 annotations across 6 protocols). The model carried over content from the one-shot exemplar and manifested 3 subtypes: drug or therapy carryover (a drug featured in the exemplar was recommended for a patient for whom it was not indicated), approval-frame carryover (the exemplar’s off-label framing caused the model to misrepresent the approval status of guideline-recommended therapies), and trial-name carryover (clinical trials cited in the exemplar were inserted in place of the patient’s actual evidence base). Multimedia Appendix 4 contains a table listing all critical errors, their types, the sections of the protocols in which they occurred, and descriptions of the deidentified content.

While language was the prominent error type for A1 (40/108, 37%), factual errors were the most prominent type in A2 (69/145, 47.6%). The median of factual errors per protocol was 0.5 (IQR 0.0‐1.0) for A1 and 1.0 (IQR 0.5‐2.0) for A2. Omission errors occurred at a median of 0.5 (IQR 0.0‐1.0) per protocol for both A1 and A2, while noise errors occurred at 0.0 (IQR 0.0‐0.5) for A1 and 0.0 (IQR 0.0‐0.0) for A2. Language errors had a median of 0.5 (IQR 0.5‐1.0) per protocol for A1 and 0.5 (IQR 0.0‐1.0) for A2.

However, median paired differences showed no notable differences in error types between A1 and A2 (factual ∆=0.0, 95% CI 0.0 to 1.5, omission ∆=0.0, 95% CI 0.0 to 0.5, and noise ∆=0.0, 95% CI 0.0 to 0.0) except for language with ∆=−0.5 (95% CI −1.0 to 0.0) while not significant.

**Figure 5.**
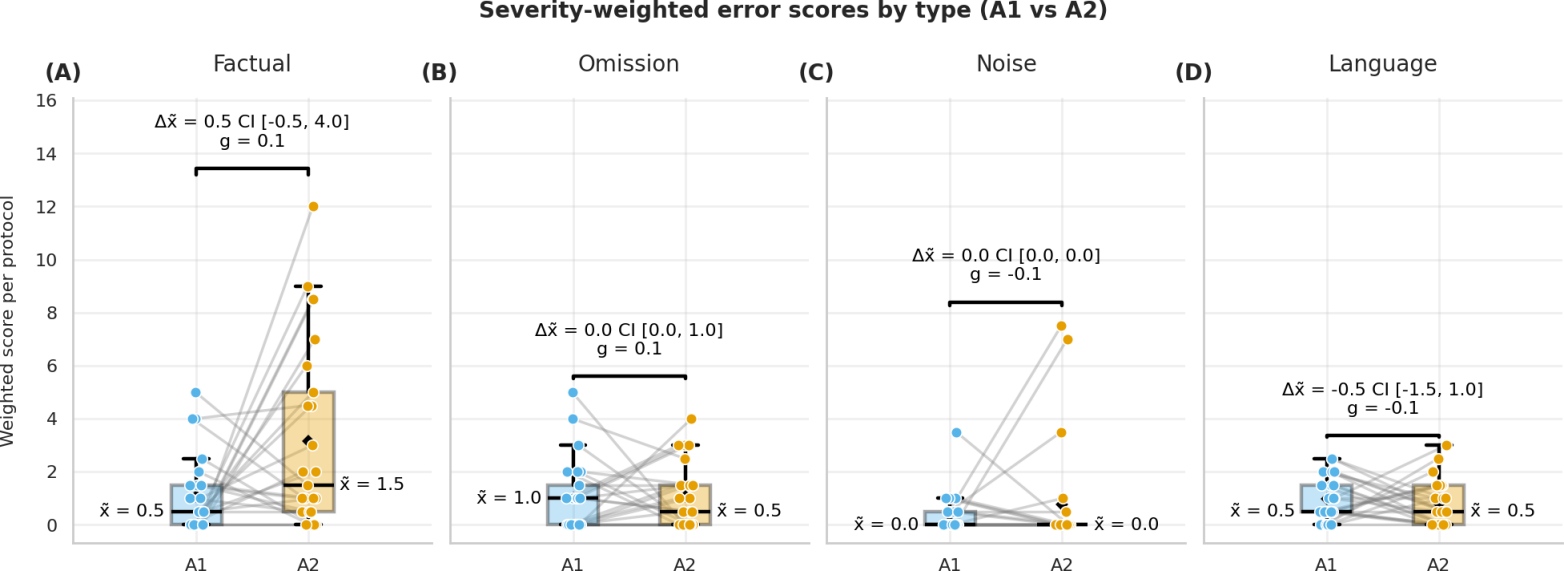
Comparison of severity-weighted error scores per protocol (minor=1, major=2, and critical=3) across prompting approaches (A1: blue and A2: orange), with annotated median (x̃), mean (diamond), median paired difference (Δx̃), its CI, and effect size of the exact binomial test. Significant differences are marked with asterisks.

Figure 5 shows the distributions of the severity-weighted error scores per protocol. It shows that for factual errors the median paired difference after severity weighting changed to ∆=0.5 (95% CI −0.5 to 4.0). For the other types, there were no notable changes when severity weighting was applied, except for the minimally increased CIs for omission and language.

**Figure 6.**
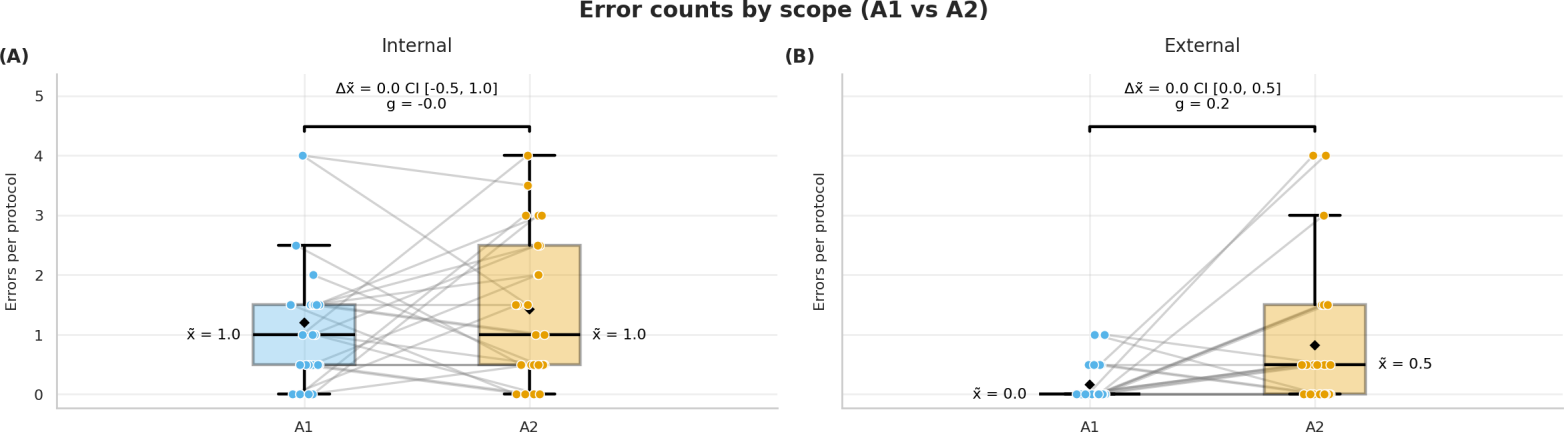
Comparison of error per protocol by scope across prompting approaches (A1: blue and A2: orange), with annotated median (x̃), mean (diamond), median paired difference (Δx̃), its CI, and effect size of the exact binomial test. Significant differences are marked with asterisks.

Looking at the scope of errors (Figure 6), internal errors dominated under both approaches, constituting 88% (60/68) under A1 and 63% (71/112) under A2. The median internal errors per protocol were 1.0 (IQR 0.5‐1.5) for A1 and 1.0 (IQR 0.5‐2.5) for A2. External errors were rare under A1 (12% of all errors; median 0.0, IQR 0.0‐0.0) but increased markedly under A2 (37% of all errors; median 0.5, IQR 0.0‐1.5).

Overall, no significant differences were detected by the exact binomial test in the error type, severity, scope, or severity-weighted error scores between A1 and A2.

The interannotator agreement analysis yielded a substantial aggregate agreement (Gwet AC1=0.72, 95% CI 0.67 to 0.76) for all errors regardless of type, severity, and scope. Almost perfect agreement [44] was reached on critical errors (Gwet AC1=0.94, 95% CI 0.92 to 0.96), which supports the validity of the critical error rate as the safety-relevant outcome of this study. Detailed reliability statistics, stratified by error type, severity, and scope, are provided in Multimedia Appendix 3.

#### Efficiency: PPEE

**Figure 7.**
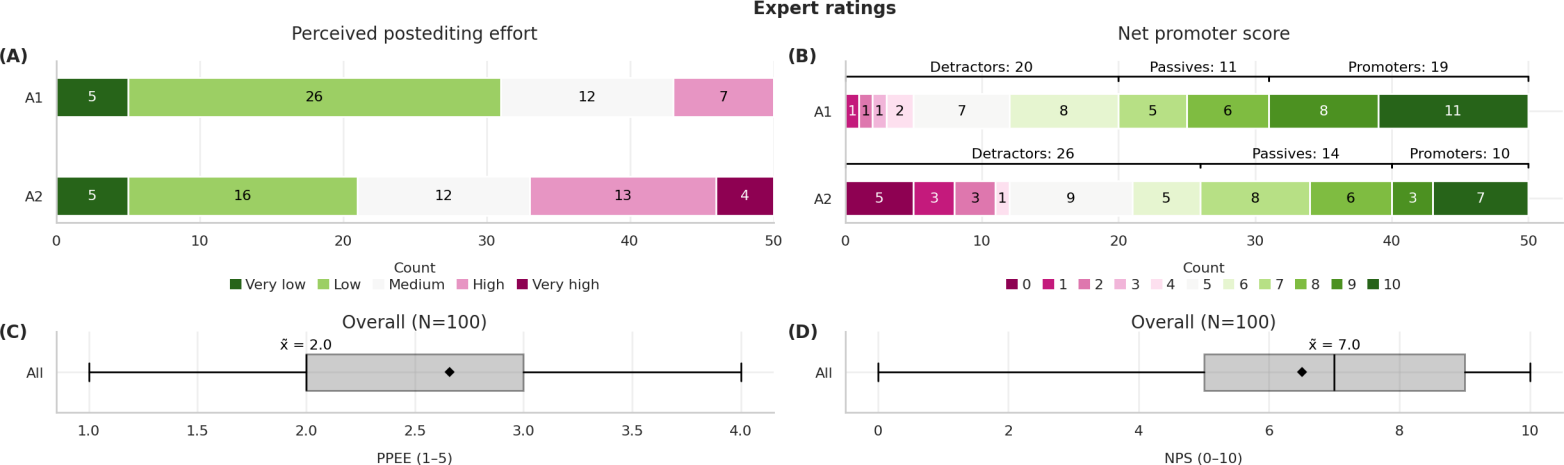
Comparison of PPEE (A and C) and NPS (B and D) ratings across prompting approaches. Green indicates better scores, and pink indicates worse scores. Boxplots of all ratings with annotated median (x̃) and mean (diamond). NPS: net promoter score; PPEE: perceived postediting effort.

Over all ratings, the PPEE (Figure 7A,C) had a median of 2.0 (95% CI 2.0 to 3.0; IQR 2.0‐3.0). A total of 52% (52/100) of ratings indicated very low to low effort, 24% (24/100) medium, and 24% (24/100) high to very high. For A1, the median was 2.5 (95% CI 2.0 to 3.0; IQR 2.0‐3.0), and for A2, the median was 3.0 (95% CI 2.5 to 3.5; IQR 2.0‐3.5). The median paired difference was *Δ*=0.5 (95% CI −0.5 to 1.0) and not significant. There was a moderate and significant (*P*<.001) correlation between PPEE and severity-weighted error score (ρ=0.55).

#### Satisfaction: NPS

Across all 100 individual ratings, the NPS (Figure 7B,D) had a median of 7.0 (95% CI 6.0 to 8.0; IQR 5‐9). The category distribution classified 29% (29/100) of ratings as promoters (scores 9‐10), 25% (25/100) as passives (scores 7‐8), and 46% (46/100) as detractors (scores 0‐6). By approach, A1 had a median of 7.0 (95% CI 6.5 to 8.5; IQR 6.0‐9.0) compared to a median of 6.5 for A2 (95% CI 4.0 to 7.0; IQR 3.5‐7.0). The detractor share rose from 40% (20/50) under A1 to 52% (26/50) under A2. Median paired differences were Δ=−1.0 (95% CI −3.0 to 0.5) and nonsignificant. Protocols rated by detractors carried a higher severity-weighted error burden, consistent with the moderate and significant (*P*<.001) correlation between NPS and severity-weighted error score (ρ=−0.54). Per rater, the detractor share ranged from 14% (2/14) to 86% (12/14), and the median item score from 4.0 (IQR 1.25-5.0) to 10.0 (IQR 10.0-10.0). The detractor predominance was concentrated in 2 raters who together contributed 24 of the 46 detractor ratings. Per-annotator distributions are reported in Multimedia Appendix 3.

## Discussion

### Principal Findings and Comparison to Prior Work

Our study evaluated the clinical usability of structured LLM-generated lay-language patient protocols under real-world conditions. While style-conditioned prompting improved automatic metrics (A2), it simultaneously increased clinically relevant error rates compared with zero-shot generation (A1).

Therefore, the most consequential finding was the systematic dissociation between automatic metric performance and expert-assessed clinical quality. This extends the critique of ROUGE and BERTScore as poorly calibrated with respect to factual consistency in general-domain abstractive summarization [45].

A2 produced statistically significant improvements in lexical fidelity, semantic alignment, and surface readability across all 8 models. The asymmetric improvement across case types (with vs without therapy recommendation) may be caused by protocols with therapy recommendation containing more dynamic fields and higher within-corpus variation. These conditions provide more surface area for style anchoring to produce measurable changes.

These findings are consistent with those of prior work, which indicate that in-context learning can improve model outputs [6]. This could suggest a preference for A2. However, expert annotation did not support this conclusion. Under A2, the proportion of protocols containing at least one critical error doubled (10/25, 40%, vs 5/25, 20%, under A1), the dominant error type shifted from language errors to factual errors, and the directional trends in both PPEE and NPS favored A1.

Although no individual comparison reached statistical significance, the consistent directional pattern across all expert-assessed outcomes (error severity, error type, postediting effort, and NPS) points to a coherent effect of prompting strategy rather than random variation. Overall, the prevalence of 20% (5/25) or 40% (10/25) of critical errors precludes the readiness of any approach for clinical use.

The mechanism underlying this dissociation is interpretable in terms of how in-context learning operates. Few-shot demonstrations primarily constrain output format and token distribution rather than conveying abstract task understanding [46]. This may lead models to generalize factual content from examples beyond their appropriate scope. In a patient-specific clinical setting, an example anchors the lexical register effectively, while simultaneously introducing factual associations that may not apply to the current protocol. In a concrete example drawn from the expert evaluation dataset, the source protocol recommended evaluation for a trial targeting their specific mutation. However, the model recommended off-label use of Olaparib, a drug not mentioned in that patient’s protocol but in the one-shot example. The protocol’s actual recommendation, trial enrollment, was omitted entirely. This drug was likely not derived from general medical knowledge, but rather was carried over from the example. Similar patterns were detected in 18 other critical errors.

Constrained decoding enforces output format but not factual content, so a schema-compliant protocol can still carry these errors. A 2-stage architecture that first extracts and verifies the clinical attributes from the source protocol [47], then generates the lay explanation from them, might mitigate this failure mode and is a direction for future work.

Model selection findings are relevant to the practical question of on-premises deployment. Llama-3.3-70B-Instruct achieved the strongest aggregate automatic metric performance and was selected for expert evaluation on this basis. However, neither parameter count nor the inclusion of reasoning capabilities was a reliable predictor of performance across metrics or models. This is consistent with early evidence that instruction-following quality is more predictive of task-specific performance than parameter scale alone [48]. This specifically matters in the European health care context where closed-source models cannot be routinely applied to real patient data under current data protection regulations without institutional data processing agreements.

Despite significant WSTF4 improvements under A2, scores across both approaches remained in the medium difficulty range (10-12), which corresponds to an approximate comprehension requirement of at least grade 10. DistilBERT-based complexity scores remained between 4.10 and 4.78 across all models and approaches, against a gold standard of 2.79. This gap highlights a clinically relevant distinction between surface readability and conceptual density. The models can simplify sentence structures without simplifying the underlying genomic concepts. This may produce protocols that read grammatically fluently yet remain incomprehensible to patients with limited health literacy. Therefore, the reviewing clinician is still responsible for bridging the conceptual gap at a level appropriate for the health literacy of each patient. The DistilBERT complexity model was fine-tuned on general German texts and may not be fully calibrated to the clinical register; therefore, the absolute gap to the gold standard should be interpreted with caution. Closing this gap will likely require domain-adapted fine-tuning, personalization to individual patient health literacy, or more granular simplification instructions that specify expected reading level and explanation depth per dynamic field.

The error type shift between approaches also has implications for the efficiency dimension of clinical usability. Linguistic errors can be identified through proofreading a single document, whereas factual errors require active cross-referencing and increased workload. Critically, undetected factual errors carry the potential for seriously harming the patient.

Expert clinicians nonetheless rated most generated drafts as requiring low to moderate PPEE. This supports the premise that structured, open-weight generation under zero-shot conditions can provide meaningful drafting support. The median NPS indicated cautiously positive satisfaction ratings overall. However, detractors outnumbered promoters, which indicates that clinician acceptance is not uniform and that a substantial proportion of clinicians remain hesitant to recommend the generated drafts to colleagues. Two factors may account for this variance. The first is the quality of the patient letter drafts, as indicated by the negative correlation between NPS and severity-weighted error scores. The second factor is inherent rater preference. The wide variation in NPS scores among raters suggests that clinicians have different thresholds for recommending AI-generated assistance to their peers, even when evaluating drafts of equivalent technical quality. Future work is required to disentangle these factors, which would necessitate a dedicated study with fuller annotator crossing and more ratings per protocol.

The clinician-supervised drafting workflow assumes that expert reviewers will detect every critical error before patient delivery, though automation bias [49] may compromise this in practice. In clinical decision support contexts, reviewers tend to over-trust AI-generated outputs and apply less scrutiny. Reviewers also perform worse under real workflow pressure. The favorable perceived effort ratings in this study may reflect this dynamic. This study did not test reviewer vigilance under realistic workflow conditions, and future work should assess error detection rates under ecologically valid review conditions before clinical deployment.

### Limitations

The specificity of MTBs constrains the generalizability of the findings of this study. MTB protocols represent a terminologically and structurally atypical genre of clinical documentation, and error profiles, readability baselines, and patient communication norms will differ substantially from other forms of documentation. The error taxonomy and evaluation framework are designed to be applicable more broadly, yet require empirical validation in other clinical contexts.

The expert evaluation was conducted with a single model (L-70B), selected on the basis of automatic metric performance. However, since this model was chosen using metrics that this study identified as imperfect proxies for clinical safety, the selection introduces a partial circularity. Therefore, the findings of the expert evaluation are conditional on this selection. Accordingly, the observed increase in critical errors under A2 is confirmed only for the L-70B model, while it is only hypothesized for other model families and institutions. Different error types or responses to prompting through the use of other models cannot be ruled out. Future work should therefore replicate the evaluation across model families. Retrieval-grounded evaluation [50] could also be explored as a complement to surface-fidelity metrics, with the potential to capture evidence-based correctness that token-level scores miss.

Regarding data representativeness, the gold-standard protocols were authored by a single expert oncologist at one institution. While the protocols were developed according to a structured framework with input from communication specialists, medical educators, and the patient advisory board, they reflect one institutional practice and one clinician’s writing style. Reference-based metrics such as ROUGE-1 and BERTScore therefore measure proximity to this single reference. They do not measure general lay-language quality. Higher scores under A2 should be read as increased stylistic alignment with the reference author, not as evidence of improved lay language quality. The underlying protocol format was therefore previously evaluated in the MyCODE study [13] and showed high patient acceptance. Nevertheless, this concentration of authorship may be a form of reference bias and should be considered when interpreting reference-based metrics.

The annotation sample of 25 cases and 50 protocols was adequate for the primary inferential analyses but had limited statistical power for subgroup comparisons, particularly for sparse error categories such as noise. The highly zero-inflated distributions required conservative sign testing in place of the Wilcoxon procedure, which may have reduced sensitivity to true directional differences between approaches. The skewed distribution of case types in expert evaluation (20 with therapy recommendation and 5 without therapy recommendation) was intentional but leaves the quality of protocols without therapy recommendation under expert scrutiny undercharacterized.

The moderate, significant correlation between severity-weighted error scores and expert ratings offers some initial evidence for the plausibility of the evaluation framework. However, expert ratings remain subjective. Future work should therefore complement them with more objective measures and assess efficiency and satisfaction through real-time usage studies rather than relying on text quality as a proxy.

Finally, all evaluation data reflect the clinician’s perspective rather than that of the intended recipient. The gold standard protocols were positively received by lay readers [13]. However, this does not extend to AI-generated output. Whether generated protocols are comprehensible and useful to patients varying in health literacy and emotional state remains an open empirical question, which the preregistered study underway (MyCODEx: DRKS00037795) is designed to address.

### Conclusions

This study evaluated structured generation of German MTB patient protocols using open-weight LLMs under on-premises deployment constraints. Zero-shot and style-conditioned one-shot prompting strategies were implemented, with performance measured via both automatic and expert clinical evaluation. Three main conclusions emerge.

First, automatic metrics and expert clinical judgment diverged systematically by approach for the expert-evaluated model. Style-conditioned one-shot prompting improved all automatic metrics across all 8 models. However, for Llama-3.3-70B-Instruct, it also doubled the rate of protocols containing at least one critical error, with the dominant error type shifting from language to factual errors. This finding suggests that reference-based metrics may be insufficient as standalone quality assurance instruments, and that expert-led evaluation is essential.

Second, neither the model parameter count nor reasoning capabilities predicted performance reliably. Under the on-premises hardware constraints required by European data protection regulations, smaller, well-calibrated models approximated the performance of substantially larger counterparts, with direct implications for sustainable and privacy-preserving deployment planning.

Third, the formative evaluation of clinical usability across all 3 ISO 9241‐11 dimensions yielded cautiously positive, yet heterogeneous results across 25 expert-evaluated cases. While overall error rates were low and efficiency ratings favorable, the near-equal split between promoters and detractors indicates that clinician acceptance is not uniform. A persistent semantic complexity gap between generated and expert-written protocols may further limit communicative adequacy for lay readers. A critical error rate of 20%‐40% precludes clinical applicability of the selected model under the conditions tested.

Taken together, structured, zero-shot LLM generation may provide a useful foundation for drafting patient-facing MTB protocols. However, these outputs require mandatory review by experts with domain expertise sufficient to verify clinical claims against the source protocol (internal) and general medical knowledge (external). The results of this study are interpretable only in an expert-supervised context, as the acceptable error threshold for clinician-reviewed drafts differs fundamentally from that required for autonomous transmission. Given the limited scale of the expert evaluation, these conclusions should be read as formative evidence informing future development rather than as a definitive clinical validation of deployment readiness.

Future work should focus on patient-centered outcome evaluation and real-world assessment of editing effort and clinical acceptance, where workflow integration will be critical for adoption. In addition, multisite validation across different clinical protocol types and the development of domain-adapted training strategies will be essential to support broader and more reliable implementation.

## Supplementary material

10.2196/99136 Multimedia Appendix 1 Model configurations and inference parameters.

10.2196/99136 Multimedia Appendix 2 Prompt templates and JSON schemas.

10.2196/99136 Multimedia Appendix 3 Detailed results, per case type analyses, agreement statistics, and additional figures.

10.2196/99136 Multimedia Appendix 4 Detailed critical error analysis.

## Abbreviations

AC1: agreement coefficient 1
AWQ: activation-aware weight quantization
BERTScore: Bidirectional Encoder Representations From Transformers Score
DistilBERT: Distilled Version of Bidirectional Encoder Representations From Transformers
ISO: International Organization for Standardization
LLM: large language model
MS: percentage of words with 3 or more syllables
MTB: Molecular Tumor Board
MXFP4: microscaling floating point 4
NPS: net promoter score
PPEE: perceived postediting effort
ROUGE: Recall-Oriented Understudy for Gisting Evaluation
SL: average sentence length in words
TRIPOD: Transparent Reporting of a Multivariable Prediction Model for Individual Prognosis or Diagnosis
WSTF4: Wiener Sachtextformel version 4
WTZ: West German Cancer Center
