# Supplementary material for: LLM-Generated Lay-Language Protocols for Molecular Tumor Board Patients: Evaluation of Quality and Clinical Usability
Source: J Med Internet Res. 2026 Jul 23;28:e99136. doi: 10.2196/99136 (PMC13397006; doi:10.2196/99136)
Supplement: Multimedia Appendix 1 [file jmir-v28-e99136-s001.docx]

### Model Configurations

The inference parameters listed in Table A1 reflect the recommended settings from each model provider's documentation. These configurations were held constant across both prompting approaches (A1 and A2) to ensure that observed performance differences could be attributed to the prompting strategy rather than to variation in decoding behavior.

**Table S1.** Inference hyperparameters for each evaluated open-weight LLM. Temperature, top_p, and top_k values were configured according to each model's official documentation. All models were served via vLLM and queried through the OpenAI Python package.

| **Model** | **temperature** | **top_p** | **top_k** |
| --- | --- | --- | --- |
| meta-llama/Llama-3.1-8B-Instruct | 0.6 | 0.9 | 0 |
| meta-llama/Llama-3.3-70B-Instruct | 0.6 | 0.9 | 0 |
| mistralai/Mistral-Large-Instruct-2411 | 1.0 | 1.0 | 0 |
| mistralai/Magistral-Small-2509 | 0.7 | 0.95 | 0 |
| openai/gpt-oss-20b | 1.0 | 1.0 | 0 |
| openai/gpt-oss-120b | 1.0 | 1.0 | 0 |
| Qwen/Qwen3-4B-Thinking-2507 | 0.6 | 0.95 | 20 |
| Qwen/Qwen3-Next-80B-A3B-Instruct | 0.7 | 0.8 | 20 |
