# Supplementary material for: LLM-Generated Lay-Language Protocols for Molecular Tumor Board Patients: Evaluation of Quality and Clinical Usability
Source: J Med Internet Res. 2026 Jul 23;28:e99136. doi: 10.2196/99136 (PMC13397006; doi:10.2196/99136)
Supplement: Multimedia Appendix 2 [file jmir-v28-e99136-s002.docx]

### Prompts

#### System Prompt

The system prompt was designed to encode institutional language standards and clinical safety constraints directly into the generation instructions. By separating structural requirements (enforced via constrained decoding) from stylistic and content rules (encoded in the prompt), the design ensures that the model operates within both formal and semantic boundaries during generation.

**Textbox S1.** System prompt used across all generation experiments. The prompt specifies the model's role as a specialist in generating German lay-friendly patient letters from MTB protocols. It defines hard constraints across four domains: language and readability (e.g., everyday vocabulary, guillemet-wrapped technical terms with lay explanations), content selection and fidelity (e.g., inclusion of only actionable findings, prohibition of fabricated therapies or references), tone and ethics (e.g., empathic and non-alarming register), and structural rules (e.g., JSON-based output following schema and field-level instructions).

| You are a specialist in generating German, lay-friendly patient letters based on molecular tumor board (MTB) protocols.  Treat all the following instructions as hard constraints and follow them exactly.  # LANGUAGE & READABILITY:  * Write in grammatically correct German and ensure it is understandable for laypeople.  * Explain all relevant information clearly and thoroughly in cohesive paragraphs.  * Use everyday vocabulary and prefer simple, commonly known synonyms over medical or technical terminology.  * If a technical term is necessary, wrap it with guillemets `» «` and give a detailed explanation in layman's terms.  * Use well-known comparisons or metaphors where possible.  * Avoid obscure language, jargon, and unnecessary conditional phrasing.  # CONTENT SELECTION & FIDELITY  * Include only information relevant to the patient and their situation.  * Include only molecular findings that directly suggest, enable, or change a therapeutic option or clinical trial eligibility.  * Exclude negative or non-actionable findings.  * Do not invent therapies, clinical trials, or literature references.  # TONE & ETHICS  * Maintain an empathic, supportive, non-alarming tone conducive to shared decision-making.  * Facilitate the doctor–patient conversation and allow the patient to review and understand the information at home.  * Avoid language that induces fear, uncertainty, or stress.  # STRUCTURAL RULES  * Construct the letter as a JSON. When the user provides a schema and field-level instructions, follow them exactly.  * Ensure the letter is cohesive and flows logically. |
| --- |

#### User Prompt Template

The template-driven approach ensures consistent prompt formatting across all protocols and schema variants (with and without therapy recommendation). The dynamic rendering of schema metadata within the prompt allows the model to receive field-level generation guidance without manual prompt authoring for each case.

**Textbox S2.** Jinja2-based user prompt template used to construct the generation instruction for each MTB protocol. The template dynamically renders field names, descriptions, enumerated options, and examples from the JSON schema, followed by the full text of the source MTB protocol enclosed in delimiters.

| Write a German patient letter based on the molecular tumorboard protocol below.  Include all these fields in your letter:{% set ordered_keys = (schema.required if (schema.required is defined and schema.required) else (schema.properties \| dictsort \| map(attribute=0) \| list)) %}{% for key in ordered_keys %}  - {{ key }}: {% if schema.properties[key].description %}{{ schema.properties[key].description }}{% endif %}{% set p = schema.properties[key] %}{% if p.enum is defined %}  - example: "{{ p.enum \| join('" \| "') }}"{% endif %}{% if p.example is defined %}  - example: "{{ p.example }}"{% endif %}{% if p.type == 'array' %}{% if p.get('items') is defined %}  - items with type: {{ p['items'].type if p['items'].type is defined else '' }}{% if p['items'].type == 'object' %}  - object fields:{% set ip = p['items'] %}{% for ikey in (ip.required if (ip.required is defined and ip.required) else (ip['properties'] \| dictsort \| map(attribute=0) \| list)) %}  - {{ ikey }}: {% if ip['properties'][ikey].description %}{{ ip['properties'][ikey].description }}{% endif %}{% endfor %}{% endif %}{% endif %}{% endif %}{% endfor %}  --- BEGIN MOLECULAR TUMORBOARD PROTOCOL ---  {{ report_text }}  --- END MOLECULAR TUMORBOARD PROTOCOL --- |
| --- |

### JSON Schemas

#### Schema for Protocols With Therapy Recommendation

This schema encodes the institutional section structure developed in collaboration with communication specialists, medical didacts, and the patient advisory board of the West German Cancer Center. By distinguishing fixed structural elements (enforced through enum values) from dynamic content fields, the schema enables constrained decoding to guarantee format compliance while preserving the model's generative flexibility for patient-specific content.

**Textbox S3**. JSON schema defining the required section structure for patient protocols derived from MTB cases with actionable therapy recommendations. The schema specifies 15 required fields including greeting, patient name, introduction, medical history, molecular findings (as a list and with individual explanations), literature summaries, therapy recommendation, closing remarks, contact person, and consultation offer. Fixed-text fields are enforced via enum constraints, while dynamic fields permit free-text generation within type and structural boundaries.

| {  "title": "Patient Letter Schema",  "description": "Schema for generating a lay-friendly letter based on a molecular tumor board protocol in structured JSON format.",  "type": "object",  "additionalProperties": false,  "required": [  "greeting",  "name",  "intro",  "medical_history",  "findings_intro",  "findings_list_start",  "findings_list",  "findings_explanations_intro",  "findings_explanations",  "literature_intro",  "literature",  "recommendation",  "closing",  "contact_person",  "consultation_offer"  ],  "properties": {  "greeting": {  "type": "string",  "description": "Appropriate greeting for the patient.",  "enum": [  "Sehr geehrter Herr",  "Sehr geehrte Frau"  ]  },  "name": {  "type": "string",  "description": "Last name of the patient."  },  "intro": {  "type": "string",  "description": "Introduction of the letter.",  "enum": [  "wie Sie wissen, haben wir Ihren Krankheitsverlauf ausführlich in unserem Molekularen Tumorboard besprochen. Wir möchten mit diesem Schreiben die Ergebnisse für Sie zusammenfassen und erklären."  ]  },  "medical_history": {  "type": "string",  "description": "Two to three sentences with lay explanations of the diagnosis and therapy history."  },  "findings_intro": {  "type": "string",  "description": "Introduction of the molecular findings section.",  "enum": [  "Um weitere therapeutische Möglichkeiten zu finden, wurde an Ihrem Tumormaterial eine molekulare Diagnostik durchgeführt. Das bedeutet, dass das genetische Profil Ihrer Tumorzellen analysiert wurde, um möglicherweise eine passgenaue Therapie für Ihre Tumorerkrankung zu finden. Man spricht in diesem Zusammenhang von einer »molekular, zielgerichteten Therapie«.", "Um weitere therapeutische Möglichkeiten zu finden, wurde an Ihrem Tumormaterial eine molekulare Diagnostik durchgeführt. Das bedeutet, dass das genetische Profil Ihrer Tumorzellen analysiert wurde. Ferner wurden auch verschiedene spezielle Oberflächenmarker auf Ihren Tumorzellen analysiert. Ziel dieser Untersuchungen war, möglicherweise eine passgenaue Therapie für Ihre Tumorerkrankung zu finden. Man spricht in diesem Zusammenhang von einer »zielgerichteten Therapie«."  ]  },  "findings_list_start": {  "type": "string",  "description": "Start of the findings list.",  "enum": [  "Im Rahmen dieser Diagnostik wurden folgende relevante Befunde erhoben:"  ]  },  "findings_list": {  "type": "array",  "description": "Short list of bullet-like strings naming the relevant molecular findings.",  "items": {  "type": "string"  },  "minItems": 1  },  "findings_explanations_intro": {  "type": "string",  "description": "Introduction of the findings explanations section that previews the detailed explanations to follow: name the findings that will be explained, note that potential therapy options will be outlined, and state which findings will not be covered and why."  },  "findings_explanations": {  "type": "array",  "description": "Multiple paragraphs with detailed lay explanations for the findings that offer therapy options as described in the intro.",  "items": {  "type": "object",  "required": [  "finding",  "explanation"  ],  "properties": {  "finding": {  "type": "string",  "description": "Name or short label of the molecular finding."  },  "explanation": {  "type": "array",  "description": "Multiple paragraphs with detailed explanations of the finding and its relevance to the patient's situation.",  "minItems": 1,  "items": {  "type": "string"  }  }  },  "additionalProperties": false  },  "minItems": 1  },  "literature_intro": {  "type": "string",  "description": "Introduction of the literature section.",  "enum": [  "Die entsprechende Datenlage möchten wir Ihnen kurz erklären:", ""  ]  },  "literature": {  "type": "array",  "description": "Multiple paragraphs with detailed lay explanations of the relevant studies and literature.",  "items": {  "type": "string"  }  },  "recommendation": {  "type": "array",  "description": "Multiple paragraphs with detailed lay explanations of the recommendation and conclusion.",  "items": {  "type": "string"  },  "minItems": 1  },  "closing": {  "type": "string",  "description": "Closing remarks.",  "enum": [  "Wir hoffen, Ihnen hiermit das Ergebnis unserer Diskussion im Molekularen Tumorboard nachvollziehbar dargestellt zu haben. Weitere Fragen besprechen Sie bitte mit"  ]  },  "contact_person": {  "type": "string",  "description": "Title and name of the treating physician(s)."  },  "consultation_offer": {  "type": "string",  "description": "Consultation offer.",  "enum": [  "Sollten darüber hinaus weitere Fragen bestehen, so stehen wir Ihren behandelnden Ärztinnen und Ärzten für einen fachlichen Austausch hinsichtlich Ihrer Erkrankung jederzeit gerne zur Verfügung."  ]  }  }  } |
| --- |

#### Schema for Protocols Without Therapy recommendation

The reduced schema reflects the inherently simpler communicative task for cases without therapy recommendations, where the primary message concerns the absence of targetable findings. The two enum variants for the findings introduction accommodate the clinical distinction between cases with no detected mutations and cases with detected but non-actionable mutations, ensuring that the patient receives an accurate characterization of their molecular diagnostic results.

**Textbox S4.** JSON schema defining the required section structure for patient protocols derived from MTB cases where no actionable molecular alterations were identified. The schema specifies 11 required fields, omitting the detailed findings list, individual findings explanations, and literature sections present in the with-therapy-recommendation schema. The findings introduction field offers two enum options addressing different molecular diagnostic outcomes (no mutations found vs. mutations found but not therapeutically actionable).

| {  "title": "Patient Letter Schema",  "description": "Schema for generating a lay-friendly letter based on a molecular tumor board protocol in structured JSON format.",  "type": "object",  "additionalProperties": false,  "required": [  "greeting",  "name",  "intro",  "medical_history",  "findings_intro",  "findings_explanations",  "recommendation_intro",  "recommendation",  "closing",  "contact_person",  "consultation_offer"  ],  "properties": {  "greeting": {  "type": "string",  "description": "Appropriate greeting for the patient.",  "enum": [  "Sehr geehrter Herr",  "Sehr geehrte Frau"  ]  },  "name": {  "type": "string",  "description": "Last name of the patient."  },  "intro": {  "type": "string",  "description": "Introduction of the letter.",  "enum": [  "wie Sie wissen, haben wir Ihren Krankheitsverlauf ausführlich in unserem Molekularen Tumorboard besprochen."  ]  },  "medical_history": {  "type": "string",  "description": "Two to three sentences with lay explanations of the diagnosis and therapy history."  },  "findings_intro": {  "type": "string",  "description": "Introductory sentences explaining the molecular findings.",  "enum": [  "Um mögliche weitere therapeutische Ansätze zu finden, wurde das genetische Profil Ihrer Tumorzellen analysiert. Dabei wurden leider keine Mutationen (Veränderungen) gefunden, die Ihre Erkrankung speziell antreiben. Das bedeutet, dass sich anhand der »molekularen Diagnostik« derzeit keine weitere Behandlungsmöglichkeit ableiten lässt.",  "Um mögliche weitere therapeutische Ansätze zu finden, wurde das genetische Profil Ihrer Tumorzellen analysiert. Dabei konnten wir zwar Mutationen - also Veränderungen - im Erbgut Ihrer Tumorzellen finden. Zum jetzigen Zeitpunkt sind diese Mutationen aber keiner zielgerichteten Therapie zugänglich. Das bedeutet, dass sich anhand der »molekularen Diagnostik« derzeit keine weitere Behandlungsmöglichkeit ableiten lässt. Die Ergebnisse unserer Untersuchungen bedeuten nicht, dass es gar keine weiteren Therapiemöglichkeiten für Sie gibt. Unsere Erklärung hier bezieht sich einzig und allein auf die Erkenntnisse, die wir aus der bisherigen molekularen Diagnostik ziehen. Bitte sprechen Sie Ihren betreuenden Onkologen darauf an."  ]  },  "findings_explanations": {  "type": "array",  "description": "Multiple paragraphs with detailed lay explanations of how many genes were tested, along with lay explanations of the results.",  "items": {  "type": "string"  },  "minItems": 1  },  "recommendation_intro": {  "type": "string",  "description": "Introduction of the recommendation section.",  "enum": [  "Da wir derzeit keine Veränderung finden konnten, für die eine »molekular zielgerichtete Therapie« zur Verfügung steht, können wir aus Sicht des Molekularen Tumorboards leider auch keine Behandlung empfehlen. Es bedeutet jedoch nicht, dass es gar keine weiteren Therapiemöglichkeiten gibt. Unsere Erklärung hier bezieht sich einzig und allein auf die Erkenntnisse, die wir aus der molekularen Diagnostik ziehen. Bitte sprechen Sie Ihren betreuenden Onkologen bzw. die Sie betreuende Onkologin darauf an.", "Da wir keine Veränderungen finden konnten, für die derzeit eine »molekular zielgerichtete Therapie« zur Verfügung steht, können wir aus Sicht des Molekularen Tumorboards auch keine Behandlung empfehlen."  ]  },  "recommendation": {  "type": "array",  "description": "Multiple paragraphs with detailed lay explanations of other recommendations besides targeted therapies.",  "items": {  "type": "string"  },  "minItems": 1  },  "closing": {  "type": "string",  "description": "Closing remarks.",  "enum": [  "Wir bedauern sehr, dass wir Ihnen derzeit keinen Ansatz für eine zielgerichtete Behandlung Ihrer Erkrankung empfehlen können. Weitere Fragen besprechen Sie bitte mit "  ]  },  "contact_person": {  "type": "string",  "description": "Title and name of the treating physician."  },  "consultation_offer": {  "type": "string",  "description": "Consultation offer.",  "enum": [  "Unabhängig davon stehen wir aber Ihren behandelnden Onkologinnen und Onkologen für einen fachlichen Austausch hinsichtlich Ihrer Erkrankung jederzeit gerne zur Verfügung."  ]  }  }  } |
| --- |

### 
