# Supplementary material for: LLM-Generated Lay-Language Protocols for Molecular Tumor Board Patients: Evaluation of Quality and Clinical Usability
Source: J Med Internet Res. 2026 Jul 23;28:e99136. doi: 10.2196/99136 (PMC13397006; doi:10.2196/99136)
Supplement: Multimedia Appendix 3 [file jmir-v28-e99136-s003.docx]

### Automatic Evaluation

#### Overall Tournament Ranking Across LLMs

The combined tournament ranking confirms that Llama-3.3-70B-Instruct (L-70B) achieved the strongest aggregate performance across all metrics and both prompting approaches, with a net win percentage of +14.1 pp. Magistral-Small-2509 (M-24B) and Mistral-Large-Instruct-2411 (M-123B) followed as the second- and third-ranked models. Notably, the two OpenAI models (G-120B and G-20B) ranked lowest despite their parameter counts and reasoning capabilities, reinforcing the finding that model size alone is not a reliable predictor of task-specific performance. This overall ranking served as the basis for selecting L-70B for the subsequent expert evaluation.


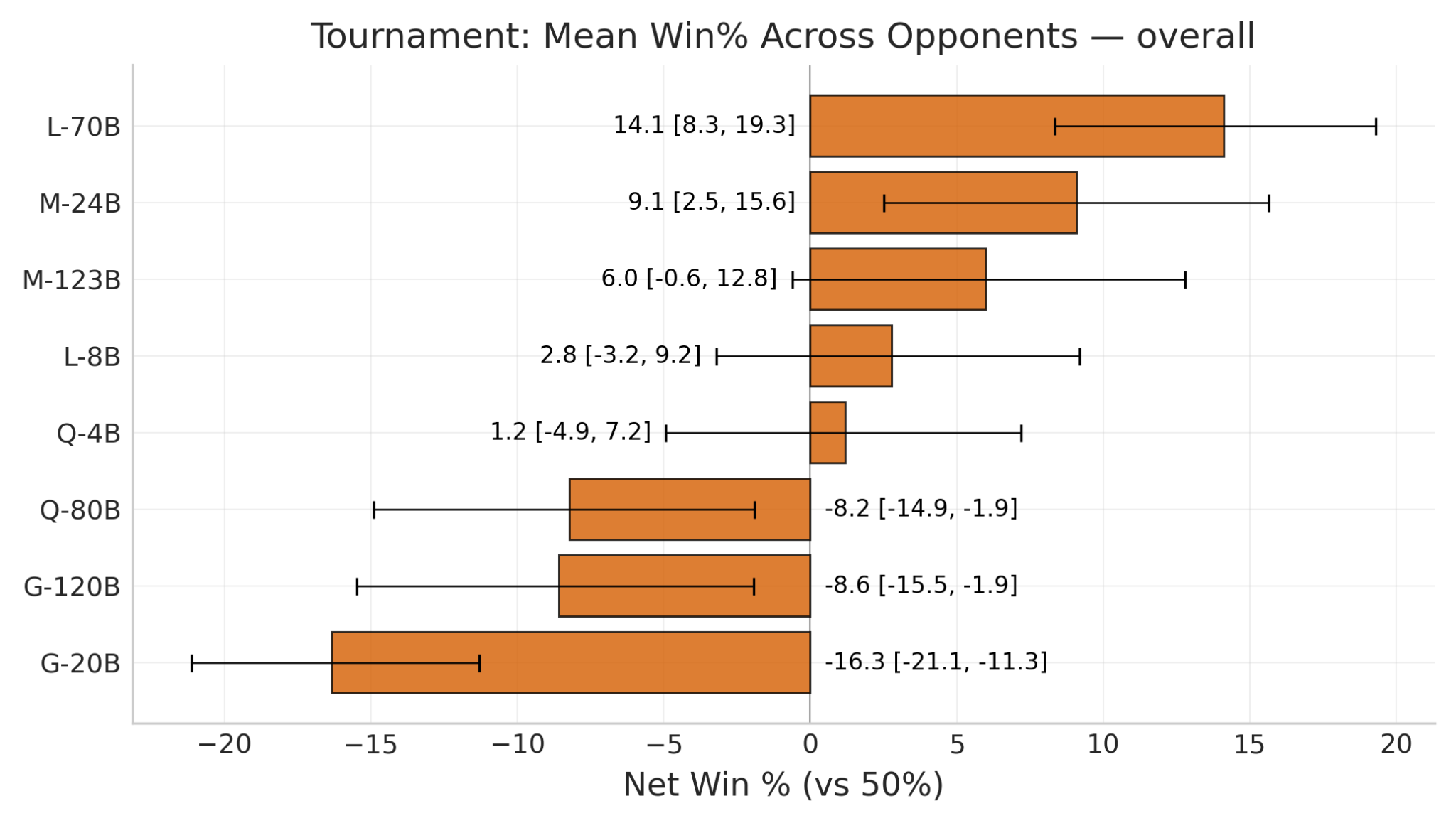


**Figure S1.** Overall tournament ranking of all eight evaluated LLMs, combining results from both prompting approaches (A1 and A2). Each bar represents the mean net win percentage of a given LLM against all other LLMs across all automatic evaluation metrics, computed relative to an overall tie baseline of 50% in pairwise comparison. Error bars indicate 95% bootstrap confidence intervals. Models are ordered by descending net win percentage.

#### Tournament Rankings by Approach and Case Type

The stratified tournament analysis reveals that model rankings were broadly consistent across case types, though some notable differences emerged. In the without-therapy-recommendation subset, Mistral-Large-Instruct-2411 (M-123B) marginally outranked Llama-3.3-70B-Instruct (L-70B) under A2, whereas L-70B maintained its top position in cases with therapy recommendation. These differences may reflect the reduced structural complexity and fewer dynamic fields in protocols without therapy recommendation, which narrows the performance range across models.


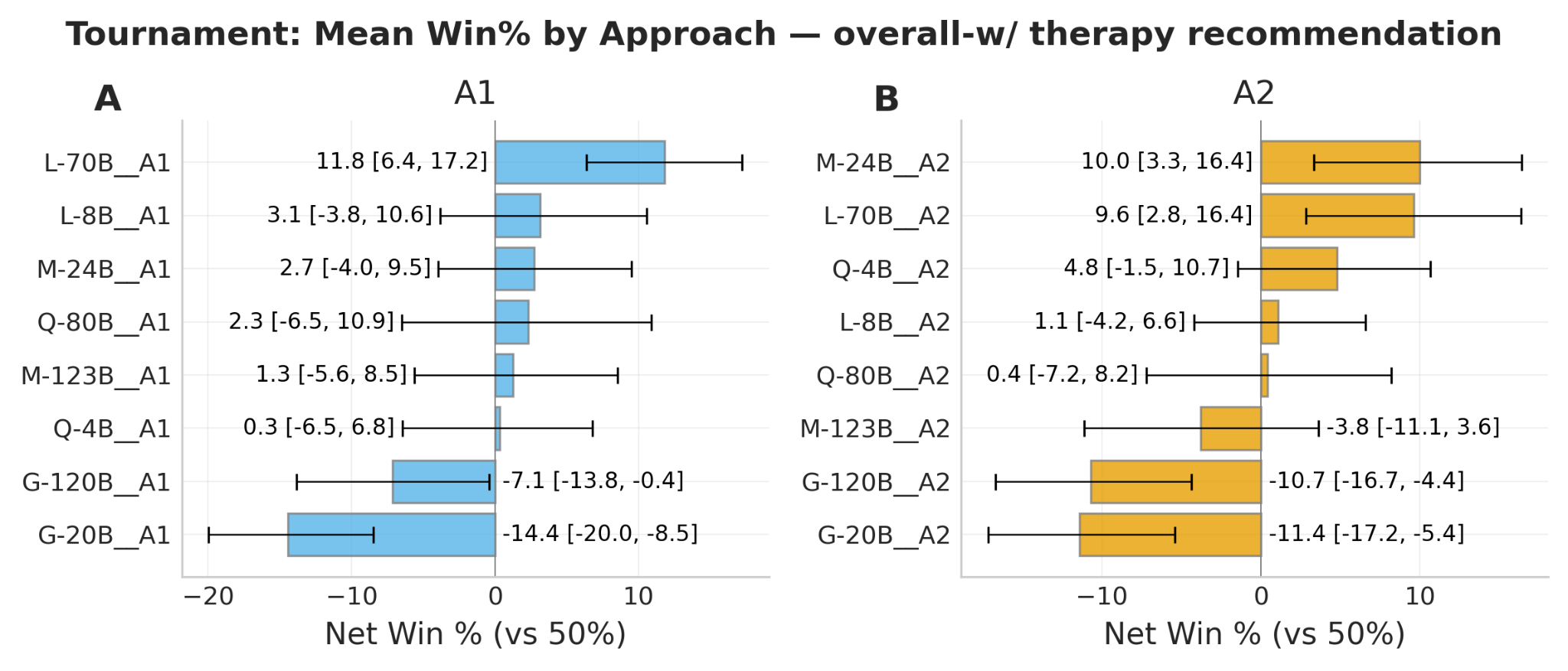


**Figure S2.** Tournament rankings stratified by case type (with therapy recommendation) for Approach 1 (A) and Approach 2 (B). Each bar represents the mean net win percentage of a given LLM against all other LLMs, computed relative to an overall tie baseline of 50% in pairwise metric comparison. Error bars indicate 95% bootstrap confidence intervals.


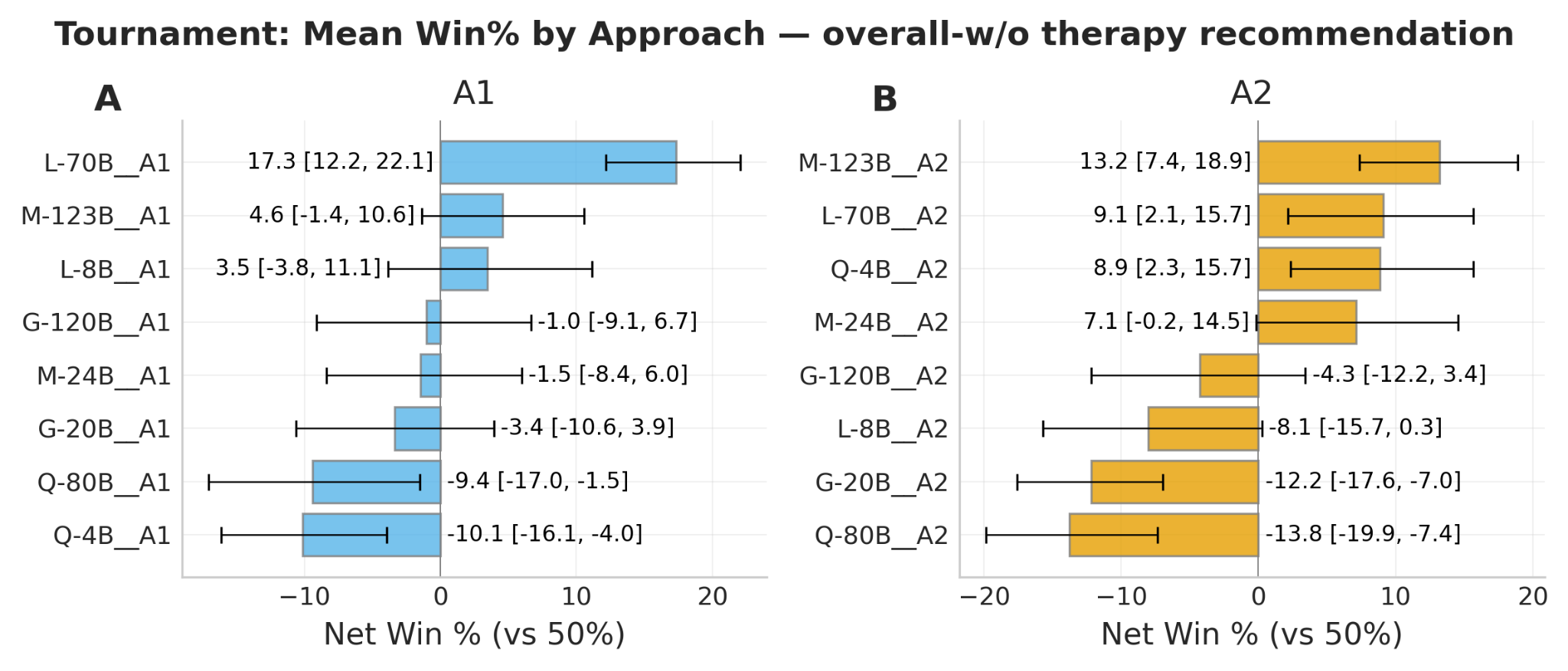


**Figure S3.** Tournament rankings stratified by case type (without therapy recommendation) for Approach 1 (A) and Approach 2 (B). Each bar represents the mean net win percentage of a given LLM against all other LLMs, computed relative to an overall tie baseline of 50% in pairwise metric comparison. Error bars indicate 95% bootstrap confidence intervals.

#### Win Proportion Matrix Comparison

The win proportion matrices complement the tournament rankings by revealing the full structure of pairwise comparisons. Notably, the matrices show that performance advantages are not uniformly distributed: some systems achieve strong aggregate rankings through consistent moderate advantages across many opponents, while others show more variable performance with larger wins against weaker systems but smaller margins against stronger ones.


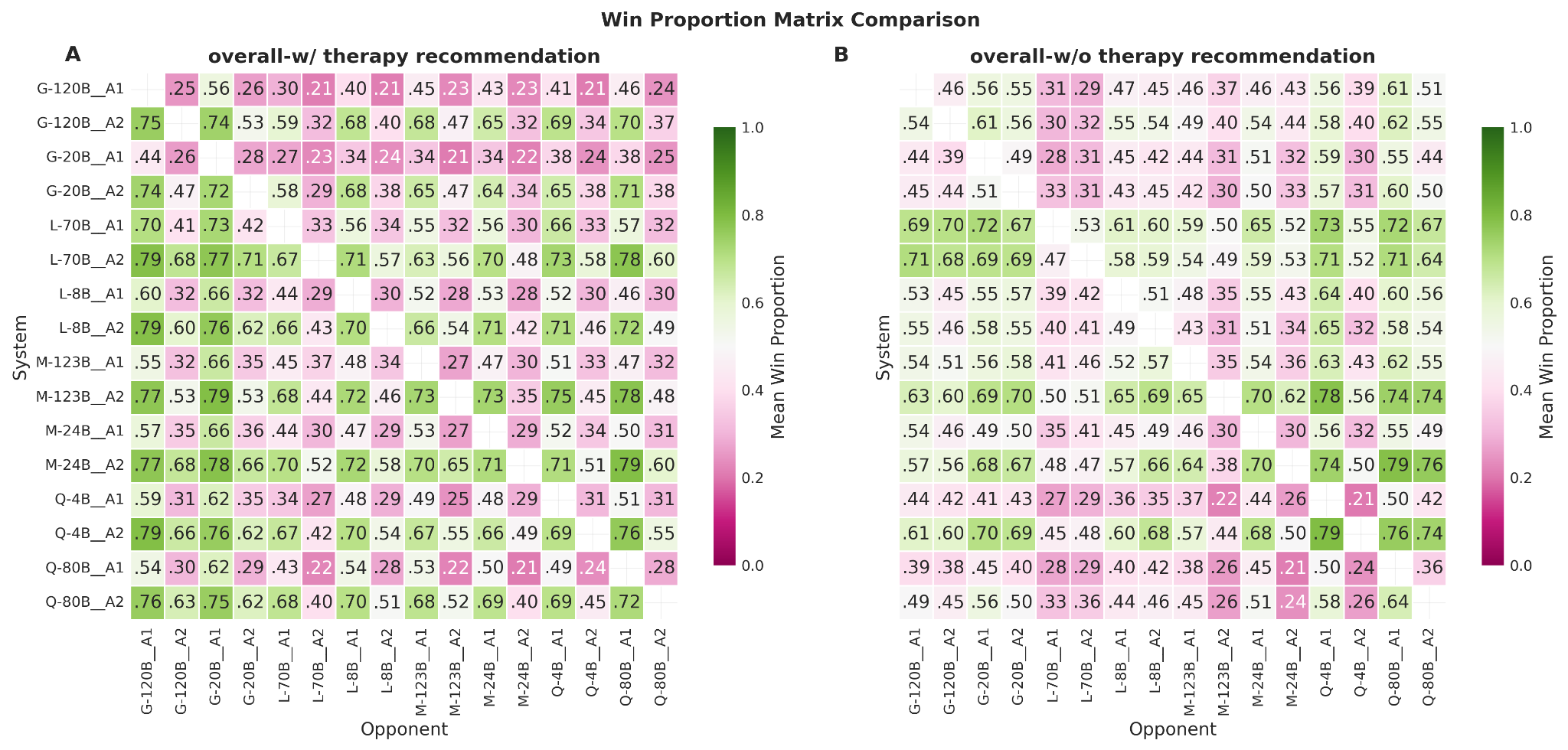


**Figure S4.** Pairwise win proportion matrices across all system combinations (LLM × approach), shown separately for protocols with therapy recommendation (panel A, left) and without therapy recommendation (panel B, right). Each cell indicates the proportion of evaluation instances in which the row system outperformed the column system across all automatic metrics. Darker shading indicates higher win proportions. The matrices provide a comprehensive view of relative system performance beyond aggregate rankings.

### Expert Evaluation

#### Effectiveness (Error Annotation)

The error type distributions reveal a qualitative shift in error profiles between approaches. Under A1, Language errors were the most frequent category, whereas under A2, Factual errors became dominant.


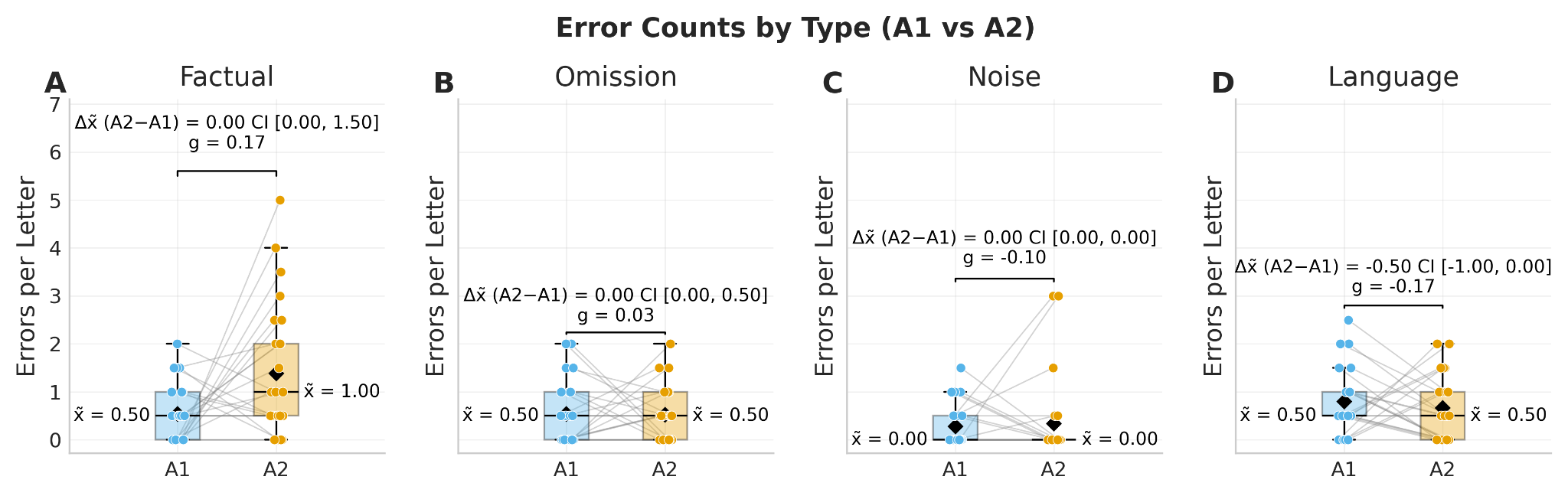


**Figure S5.** Comparison of error counts per protocol by error type across prompting approaches (A1: blue, A2: orange). Panel A shows Factual errors, Panel B shows Omission errors, Panel C shows Noise errors, and Panel D shows Language errors. Each plot displays individual data points (jittered), boxplots with annotated median (x̃), mean (diamond marker), median paired difference (Δx̃), its 95% bootstrap confidence interval, and the effect size (Cohen's g) from the exact binomial sign test.

The severity-level analysis shows that while minor errors occurred at comparable rates across both approaches, the trends for major and critical errors pointed toward higher rates under A2.


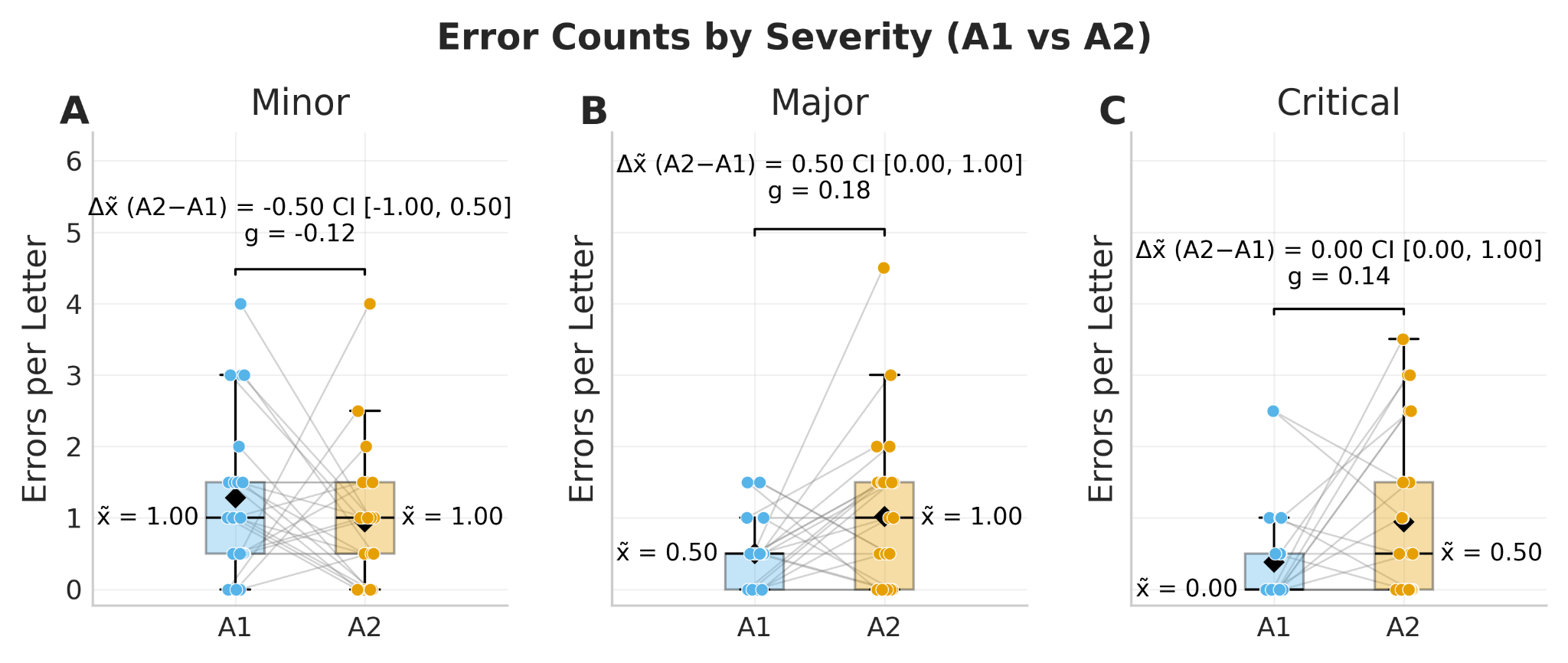


**Figure S6.** Comparison of error counts per protocol by severity level across prompting approaches (A1: blue, A2: orange). Panel A shows Minor errors, Panel B shows Major errors, and Panel C shows Critical errors. Each plot displays individual data points (jittered), boxplots with annotated median (x̃), mean (diamond marker), median paired difference (Δx̃), its 95% bootstrap confidence interval, and the effect size (Cohen's g) from the exact binomial sign test.

This cross-tabulation provides the most granular view of error profile differences between approaches. The most notable shifts include increased frequencies of external factual errors (particularly major and critical) under A2, and decreased frequencies of minor language errors.


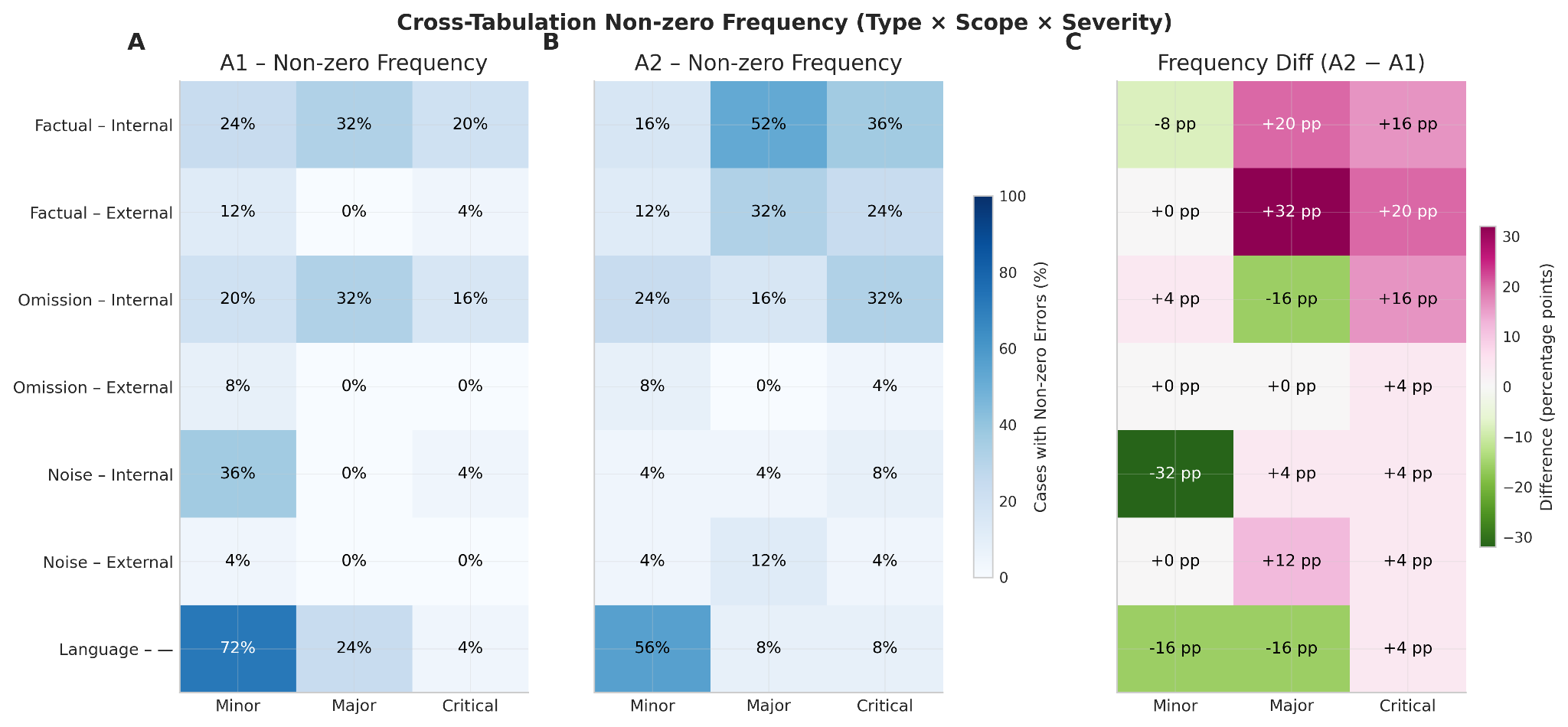


**Figure S7.** Cross-tabulation of non-zero error frequencies across the full taxonomy (Type × Scope × Severity) for A1 (panel A) and A2 (panel B), with frequency differences (A2 − A1) shown in panel C. Each cell reports the percentage of protocols containing at least one error of the corresponding type–scope–severity combination. Panel C visualizes the direction and magnitude of frequency shifts between approaches, stratified by severity level (Minor, Major, Critical).

#### Inter-annotator Agreement

Agreement was substantial to almost perfect across all analysis categories when assessed with Gwet's AC1, ranging from 0.72 (all errors) to 0.96 (Noise only). As expected, Krippendorff's alpha values were considerably lower, particularly for sparse error categories such as Omission (α = 0.05) and Minor errors (α = 0.13), illustrating the prevalence paradox that motivates the use of AC1 in imbalanced annotation settings. The high AC1 values for critical errors (0.94) are particularly relevant, as reliable identification of safety-critical issues is a prerequisite for the clinical application of the evaluation framework.

**Table S1.** Inter-annotator agreement statistics for error annotation, stratified by severity level (Critical, Major, Minor), error type (Factual, Omission, Noise, Language), and scope (Internal, External). Metrics include observed agreement, prevalence index, Krippendorff's alpha and Gwet's AC1 with their 95% bootstrap confidence intervals. AC1 was selected as the primary reliability measure due to the highly imbalanced nature of the data.

| **Analysis** | **Agree-ment** | **Preva-lence** | **Krippen-dorff’s α** | **α CI Low** | **α CI High** | **Gwet’s AC1** | **AC1 CI Low** | **AC1 CI High** |
| --- | --- | --- | --- | --- | --- | --- | --- | --- |
| All | 0.80 | 0.64 | 0.33 | 0.25 | 0.42 | 0.72 | 0.67 | 0.76 |
| Critical | 0.94 | 0.90 | 0.39 | 0.24 | 0.54 | 0.94 | 0.92 | 0.95 |
| Major | 0.89 | 0.86 | 0.15 | 0.04 | 0.26 | 0.87 | 0.84 | 0.90 |
| Minor | 0.86 | 0.82 | 0.13 | 0.03 | 0.23 | 0.83 | 0.79 | 0.86 |
| Factual | 0.90 | 0.85 | 0.23 | 0.11 | 0.35 | 0.88 | 0.85 | 0.91 |
| Omission | 0.90 | 0.89 | 0.05 | -0.04 | 0.15 | 0.89 | 0.86 | 0.91 |
| Noise | 0.96 | 0.95 | 0.26 | 0.06 | 0.46 | 0.96 | 0.95 | 0.98 |
| Language | 0.91 | 0.88 | 0.17 | 0.05 | 0.29 | 0.90 | 0.87 | 0.92 |
| Internal | 0.83 | 0.70 | 0.31 | 0.20 | 0.41 | 0.77 | 0.71 | 0.82 |
| External | 0.93 | 0.91 | 0.25 | 0.08 | 0.42 | 0.93 | 0.90 | 0.95 |

#### Efficiency (Perceived Post Editing Effort) and Satisfaction (Net Promoter Score)

Both efficiency and satisfaction metrics showed directional trends favoring A1 over A2, though neither comparison reached statistical significance. The NPS median was 7.00 for A1 versus 6.50 for A2, and the PPEE median was 2.50 for A1 versus 3.00 for A2. These trends are consistent with the higher rate of critical and factual errors observed under A2, suggesting that error profiles influence clinician perceptions of both editing burden and overall recommendation likelihood.


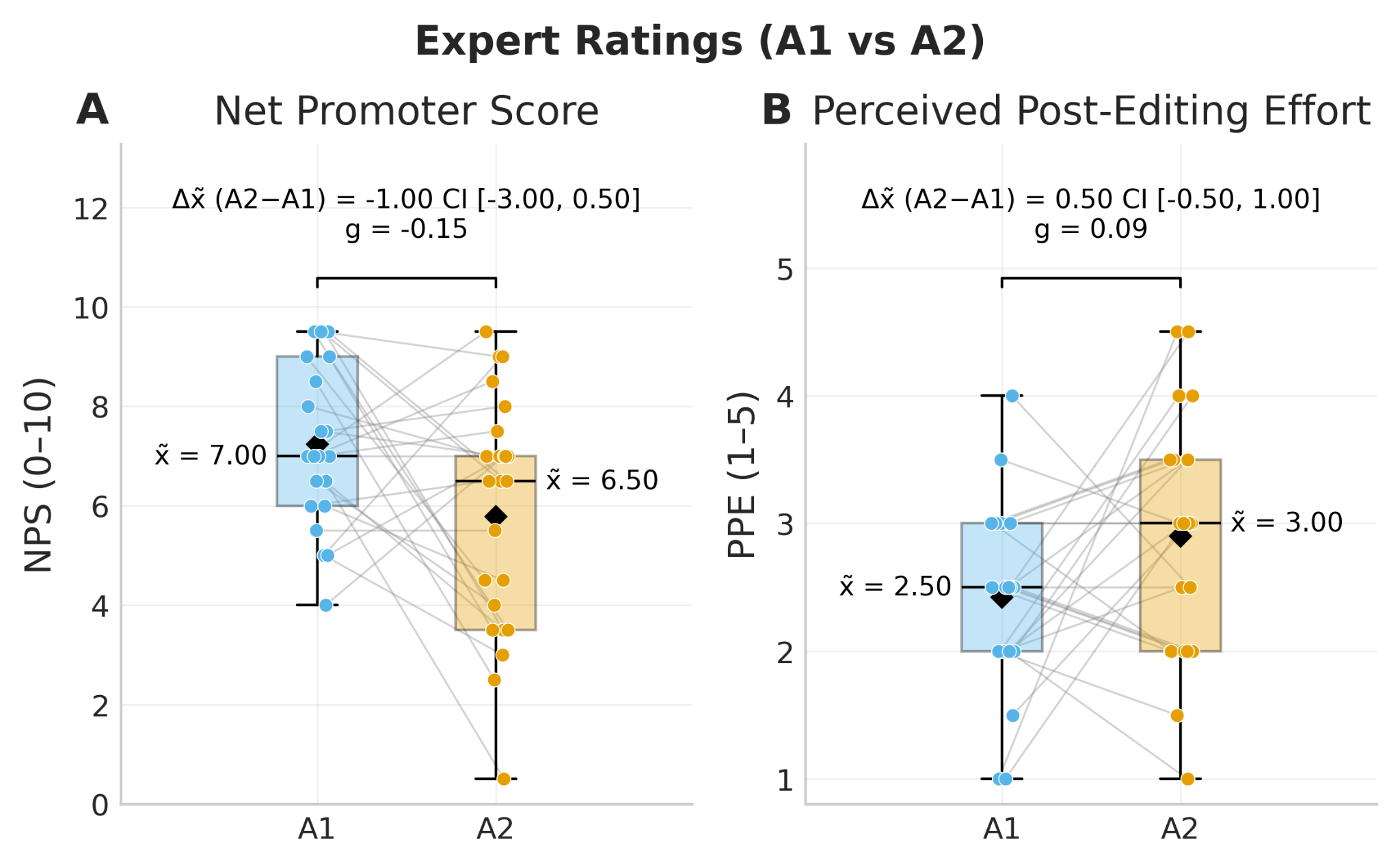


**Figure S8.** Comparison of expert ratings across prompting approaches for Net Promoter Score (NPS, panel A) and Perceived Post-Editing Effort (PPEE, panel B). Boxplots show the distribution of case-level median ratings with annotated median (x̃), mean (diamond marker), median paired difference (Δx̃), its 95% bootstrap confidence interval, and the effect size (Cohen's g) from the exact binomial sign test.


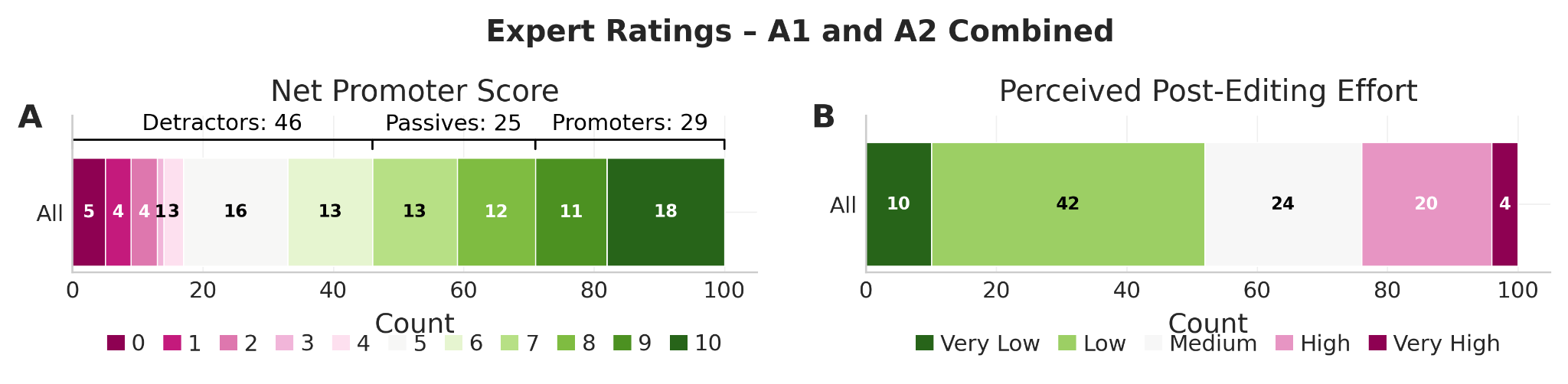


**Figure S9.** Distribution of all expert ratings (N = 100) across both prompting approaches combined. Panel A shows the Net Promoter Score distribution with standard NPS classification (Detractors: scores 0–6, Passives: scores 7–8, Promoters: scores 9–10). Panel B shows the Perceived Post-Editing Effort distribution across the five-point Likert scale (Very Low to Very High).

The combined rating distributions reveal meaningful heterogeneity in clinician responses. Detractors (46%) substantially outweighed Promoters (29%) on the NPS, indicating that a significant proportion of clinicians remain hesitant to recommend AI-generated drafts to colleagues. For PPEE, the majority of ratings (52%) fell in the very low to low range, suggesting that most generated protocols were perceived as requiring modest editing effort to reach patient-ready quality, despite the presence of errors identified through systematic annotation.

**Table S2.** Per-annotator distribution of recommendation-likelihood ratings across the three Net Promoter Score categories (Detractors: 0–6, Passives: 7–8, Promoters: 9–10) and median item score. Rater labels are anonymized. Percentages are based on each annotator's individual rating count.

| **Annotator** | **Ratings n** | **Promoters n (%)** | **Passives n (%)** | **Detractors n (%)** | **Median** |
| --- | --- | --- | --- | --- | --- |
| R1 | 14 | 12 (86%) | 0 (0%) | 2 (14%) | 10.0 |
| R2 | 14 | 8 (57%) | 1 (7%) | 5 (36%) | 9.0 |
| R3 | 14 | 0 (0%) | 2 (14%) | 12 (86%) | 4.0 |
| R4 | 15 | 0 (0%) | 9 (60%) | 6 (40%) | 7.0 |
| R5 | 14 | 5 (36%) | 4 (29%) | 5 (36%) | 7.0 |
| R6 | 14 | 4 (29%) | 6 (43%) | 4 (29%) | 8.0 |
| R7 | 15 | 0 (0%) | 3 (20%) | 12 (80%) | 6.0 |

#### Correlation of Severity-Weighted Error Score and Expert Ratings

The moderate correlations between severity-weighted error scores and both NPS and PPEE provide initial evidence for plausibility of the evaluation framework: protocols with more severe errors received lower satisfaction ratings and higher perceived editing effort. The consistent directionality across both expert-rated outcomes supports the use of the proposed error taxonomy as a meaningful indicator of clinical usability. Notably, A2 data points indicate higher error scores and less favorable expert ratings, visually reinforcing the approach-level findings.


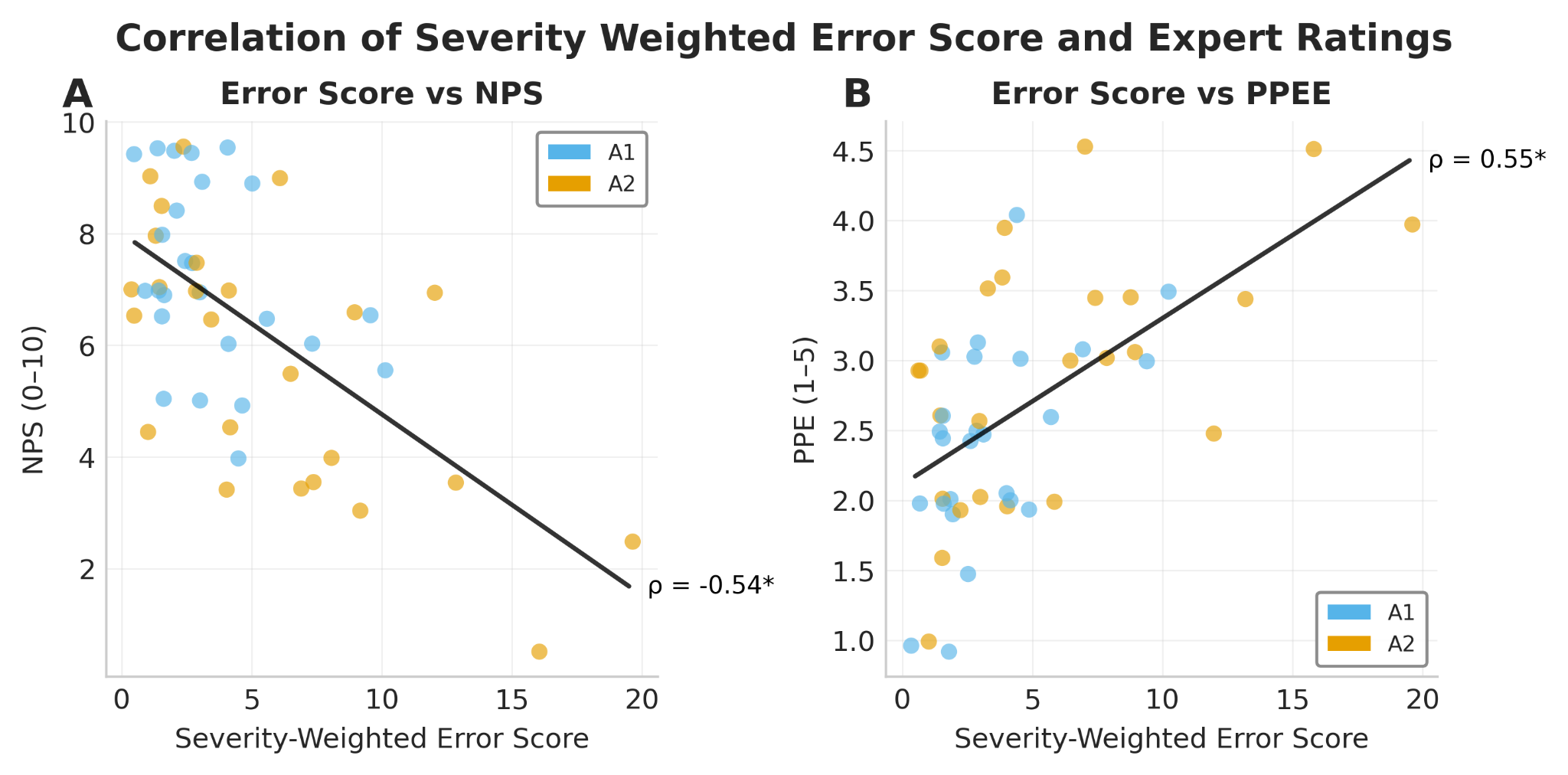


**Figure S10.** Scatter plots showing the relationship between severity-weighted error scores and expert ratings. Panel A shows the correlation with NPS (Spearman's ρ = −0.54, P < .001), and Panel B shows the correlation with PPEE (Spearman's ρ = 0.55, P < .001). Data points are colored by prompting approach (A1: blue, A2: orange).
